# Additive-driven microwave crystallization of tyramine polymorphs and salts: a quantum crystallography perspective

**DOI:** 10.1107/S2052252525002210

**Published:** 2025-04-28

**Authors:** Szymon Grabowski, Klaudia Nowakowska, Helena Butkiewicz, Anna Hoser, Aleksandra Wesełucha-Birczyńska, Tomasz Seidler, Paulina Moskal, Marlena Gryl

**Affiliations:** a Faculty of Chemistry Jagiellonian University Gronostajowa 2 Krakow30-387 Poland; b Doctoral School of Exact and Natural Sciences Jagiellonian University Prof. St. Łojasiewicza 11 Krakow30-348 Poland; c Biological and Chemical Research Centre Faculty of Chemistry University of Warsaw Żwirki i Wigury 101 Warsaw02-089 Poland; Universidad de Oviedo, Spain

**Keywords:** tyramine, polymorphism, microwave cocrystallization, electron density studies, optical properties

## Abstract

Polymorphism – the ability of a compound to exist in multiple crystalline forms – needs to be carefully considered in the design of functional materials, particularly in the context of cocrystallization. Tyramine, a biogenic amine, is a promising candidate for polymorph exploration due to its conformational flexibility and ability to form salts. In this study, we investigate the crystallization of tyramine polymorphs using additives and microwave-assisted techniques. Our findings reveal the formation of a new tyramine polymorph and two distinct salts, highlighting the impact of microwave radiation and additive-driven crystallization on polymorph stability and molecular encapsulation. The study demonstrates that the triclinic tyramine polymorph (T2) is thermodynamically more stable due to its lower electronic energy, whereas the monoclinic form (T1) features slightly stronger intermolecular interactions. Over time, in solution, crystals of barbital–tyramine salts (C1 and C2) begin to form, providing an opportunity to assess structural evolution. Optical properties calculations show significant maximum linear birefringence values (0.164 and 0.255) for two polymorphs of tyramine, whereas for C1, this value decreases to 0.095.

## Abstract 2

This study reveals how additives and microwave radiation influence the crystallization of new tyramine polymorphs and their cocrystallization with barbital. The findings provide insights into polymorph stability and offer potential applications in molecular encapsulation and optical materials.

## 1. Introduction

The phenomenon of polymorphism, defined as the ability of a chemical compound to exist in more than one crystalline form, plays a critical role in the design of functional materials. Molecules susceptible to polymorphism are valuable in cocrystallization due to their intrinsic adaptability to new interaction environments. Creating multicomponent materials, such as co-crystals or salts, by predicting possible intermolecular interactions (Sun *et al.*, 2021 ▸) is one of the ways to modify and control the physical properties of materials. Polymorphs, despite having the same chemical composition, often exhibit different properties, such as solubility, dissolution rate, chemical and mechanical stability, or bioavailability – an important factor in medical sciences (Saifee *et al.*, 2009 ▸). In addition, they frequently differ in crystal symmetry, which is fundamental to the manifestation of certain physical properties, especially those associated with chiral or polar molecular arrangements, *e.g.* pyro- and ferroelectricity, optical activity and many more (Newnham, 2005 ▸). Consequently, a thorough understanding and precise control of polymorphism are essential for enhancing the performance and expanding the applications of crystalline materials.

Over the years an unconventional approach towards crystallization and cocrystallization has gained much attention as it can offer advantages such as enhanced control over crystal properties, faster crystallization rates and the ability to obtain unique crystal forms that are difficult to achieve through traditional methods. These methods often deviate from classical crystallization practices, which typically involve slow evaporation or cooling of a solution. Unconventional methods can include the use of alternative energy sources, novel solvents and innovative techniques to influence nucleation and crystal growth. Examples include microwave-assisted crystallization (Rodrigues *et al.*, 2020 ▸), sonocrystallization (Evrard *et al.*, 2020 ▸), electrocrystallization (Wan *et al.*, 2023 ▸) or supercritical fluid crystallization (Qiao *et al.*, 2020 ▸). Additionally, methods involving usage of additives and seeding processes are becoming exceedingly popular (Kitamura, 2009 ▸; Davey *et al.*, 1997 ▸; Weissbuch *et al.*, 2003 ▸; Lévesque *et al.*, 2020 ▸). The role of additives can be attributed to their effects on solubility or surface tension during nucleation (Xu *et al.*, 2022 ▸). Many new polymorphic forms were obtained using additives, examples include the influence of amino acids on the crystallization of l-glutamic acid, where they stabilize the α-form (Cashell *et al.*, 2005 ▸), and the control of crystallization of forms I and II of 5-fluoro­uracil by varying the amount of nicotinamide in solution (Enkelmann *et al.*, 2019 ▸). The impact of microwave radiation on the crystallization process can also be significant, as it increases the molecules’ rotational freedom, which can lead to nontypical mutual reorientation (Pagire *et al.*, 2013 ▸). There are reports in the literature of new co-crystal formations in the presence of microwave radiation during the crystallization process, such as caffeine/maleic acid (Pagire *et al.*, 2013 ▸), loratadine/DIMEB (Nacsa *et al.*, 2008 ▸), sulfa-drug co-crystals (Ahuja *et al.*, 2020 ▸) and caffeic acid phenethyl co-crystals (Ketkar *et al.*, 2016 ▸).

**Figure 1:**
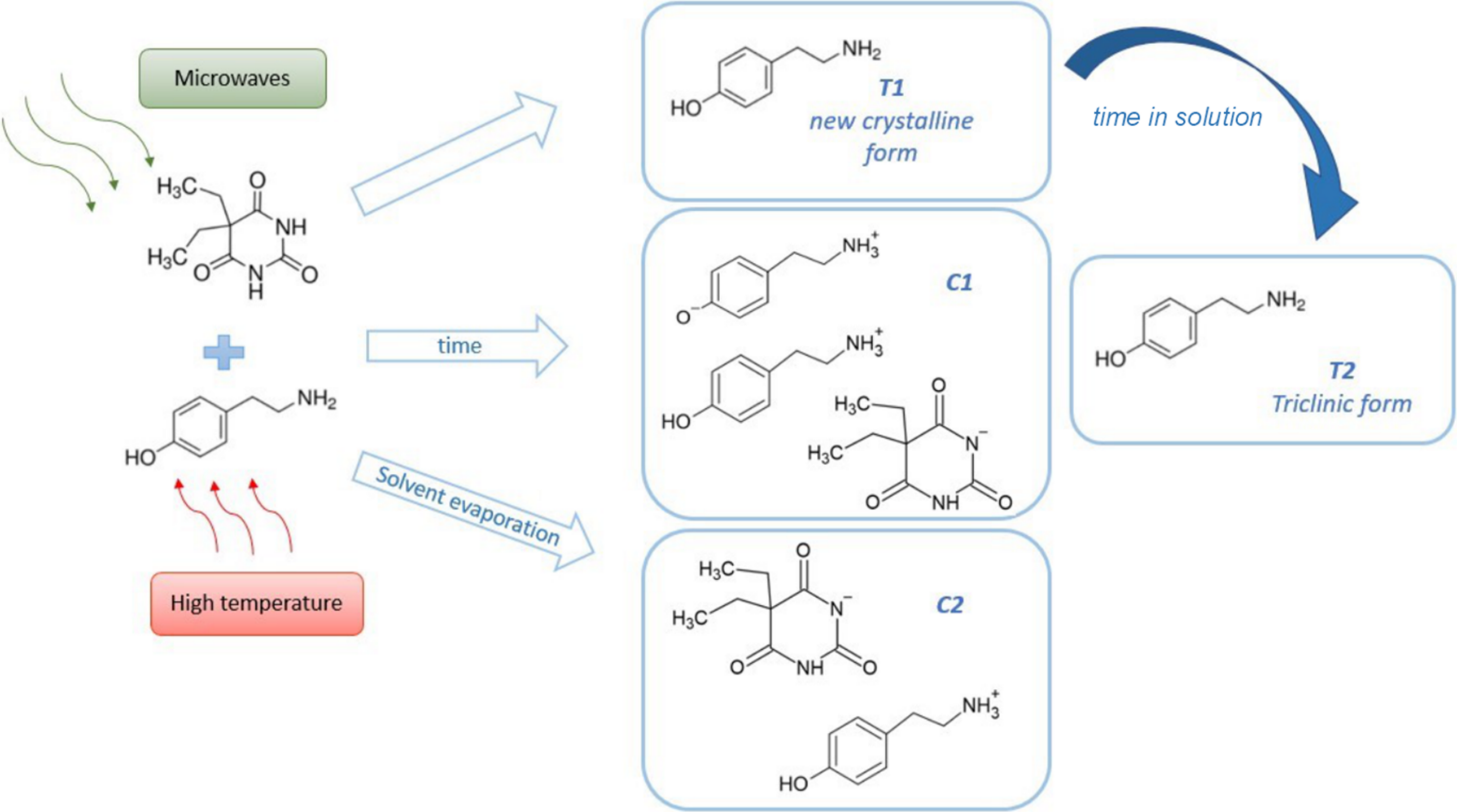
Cocrystallization of tyramine and barbital using microwaves with all possible products.

In this work, we examine the influence of an additive and the impact of microwave radiation on the crystallization of tyramine polymorphic forms. The additive barbital not only facilitates the formation of a previously unknown tyramine polymorph, but over time two distinct salts with tyramine also emerge in the crystallization batch, reflecting the evolution of the system (Fig. 1 ▸).

Tyramine [4-(2-amino­ethyl)­phenol] is a biogenic amine occurring in some natural products, famous for its dangerous interaction with drugs called the ‘cheese effect’ (Sadighara *et al.*, 2024 ▸). This condition refers to the hypertensive crisis that can occur when consuming tyramine-rich foods, like certain cheeses and fermented products, while taking mono­amine oxidase inhibitors (MAOIs). Normally, mono­amine oxidase enzymes break down excess tyramine in the body. However, when MAOIs are used as medications, they inhibit the activity of these enzymes, leading to elevated levels of tyramine. This increase in tyramine can cause a rapid and dangerous rise in blood pressure, known as a hypertensive crisis. Symptoms of a hypertensive crisis include severe headache, palpitations, nausea, sweating and, in severe cases, stroke or heart attack (Anderson *et al.*, 1993 ▸; McCabe-Sellers *et al.*, 2006 ▸).

In crystal engineering, tyramine is a promising component for obtaining new organic and inorganic salts. This potential was demonstrated by Briggs *et al.* (2012 ▸), who described 42 salt forms of the compound. This finding is supported by the number of tyramine salts documented in the Cambridge Structural Database (CSD) (Briggs *et al.*, 2012 ▸; Rydz *et al.*, 2018 ▸; Cruickshank *et al.*, 2013 ▸; Gryl *et al.*, 2019 ▸; Kolev *et al.*, 2009 ▸; Ishida & Inoue, 1981 ▸; Prohens *et al.*, 2014 ▸; Parveen *et al.*, 2017 ▸; Parveen & Dastidar, 2016 ▸, 2015 ▸; Ohba & Ito, 2002 ▸; Nguyen *et al.*, 1998 ▸; Mittapalli *et al.*, 2019 ▸; Ivanova, 2012 ▸) – 63 structures as of the publication date. This diversity is likely due to the conformational flexibility of the aliphatic chain, which can either extend (torsion angle approximately ±170°) or fold towards the aromatic ring (torsion angle approximately 60–70°), and its ability to form salts (p*K*_a_) and hydrates (Briggs *et al.*, 2012 ▸; Andersen, 1977 ▸). Surprisingly, to date, only one polymorphic form of tyramine has been identified in the CSD.

We hypothesized that, due to its conformational flexibility, synthon formation ability and capacity to form hydrates, tyramine is capable of forming more than one polymorphic modification. As such and considering all the above-mentioned interesting properties of tyramine, we decided to investigate it more thoroughly. In 2019, we obtained two new solvates of tyramine barbitalate (Rydz *et al.*, 2018 ▸). Both phases are characterized by large unit cells (*V* up to 16000 Å^3^) and contain rare barbitalate anions. However, their crystal structures differed dramatically. The trigonal form exhibited an 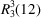 motif, responsible for the presence of voids filled with disordered solvent molecules (water and ethanol). In contrast, the monoclinic form contained four tyramine ions, four barbital ions and four chloro­form molecules in the asymmetric unit (*Z*′ = 4). We speculated whether the high-symmetry form could be maintained without the solvent; however, heating the sample to 135°C caused the collapse of the crystal structure, resulting in recrystallization of one of the barbital polymorphs [5,5-di­ethyl­pyrimidine-2,4,6-(1*H*,3*H*,5*H*)-trione].

In order to investigate whether we can entrap other small molecules in the cages, we proposed an experiment exchanging the solvent with another that is small enough to be retained in the voids, thereby attempting to obtain an isostructural architecture similar to the previously examined one. Utilizing microwave-assisted crystallization, we successfully obtained a new, noncentrosymmetric, tyramine crystal structure (*Pc*) and two salts of tyraminium barbitalate: one containing water molecules and the other aceto­nitrile, with the aceto­nitrile salt maintaining the trigonal architecture. As these phases formed sequentially, we could monitor the evolution of the tyramine–barbital system. We examined the crystal phases obtained using hot-stage microscopy, DSC, Raman spectroscopy and quantum crystallography tools to understand the formation of these crystal phases and the transformation processes within this complex system. Also, as the new tyramine polymorph is polar with a promising orientation of tyramine molecules, our focus turned to (non)linear optical properties. The aim of this paper is to explain the role of additives in the crystallization of tyramine polymorphs, the impact of microwave radiation and the influence of polymorph stability on the cocrystallization process of tyramine–barbital salts. This study evaluates the potential of tyramine–barbital salts for entrapping small molecules and hence assesses their utility in molecular encapsulation.

## 2. Experimental

### 2.1. Crystallization

50 mg of tyramine (0.36 mmol) and 66 mg of barbital (0.36 mmol) were dissolved in 6 ml of aceto­nitrile. The synthesis was performed in a microwave reactor, heating the sample for 5 min at 150°C and then cooling it to 70°C. The resulting clear, colourless solution was allowed to crystallize slowly. After 1.5 h, colourless crystals (T1) formed as thin plates. The product was left in the mother liquor, and after a couple of days the crystals had turned slightly yellow, indicating the formation of a tyramine–barbital salt co-crystal (C1). After the solvent had almost completely evaporated, large orange single crystals were observed at the bottom of the beaker, indicating the formation of a second tyraminium barbitalate salt (C2). The cocrystallization process and the molecular graphs of the substrates and products are presented in Fig. 1 ▸.

### 2.2. Single-crystal X-ray diffraction – data collection and refinement

The most suitable single crystals of T1, T2, C1 and C2 for X-ray diffraction analysis were selected using a polarizing microscope. Measurements were performed on a Rigaku Synergy S at appropriate temperatures and using a given radiation type (details are given in Table S1 of the supporting information). For T1, additional measurements were performed using Cu *K*α radiation at 243, 234, 226, 222, 203 and 170 K, but in all cases there were no distinct differences in the data. Final data for T1 and T2 were collected at 100 K using Mo *K*α radiation to achieve better resolution. Data reduction and integration were performed with *CrysAlisPro* (Rigaku, 2018 ▸). Crystal structures were solved using *SHELXT* (Sheldrick, 2015*b* ▸) and refined using *SHELXL*-2018/3 (Sheldrick, 2015*a* ▸). For all structures the hydrogen atoms connected to aromatic or aliphatic carbon atoms were placed in idealized positions and included in the refinement using the riding model. The remaining hydrogen atoms bonded to nitro­gen and oxygen were located on difference Fourier maps, and their positions were subsequently refined.

### 2.3. X-ray powder diffraction

**Figure 2:**
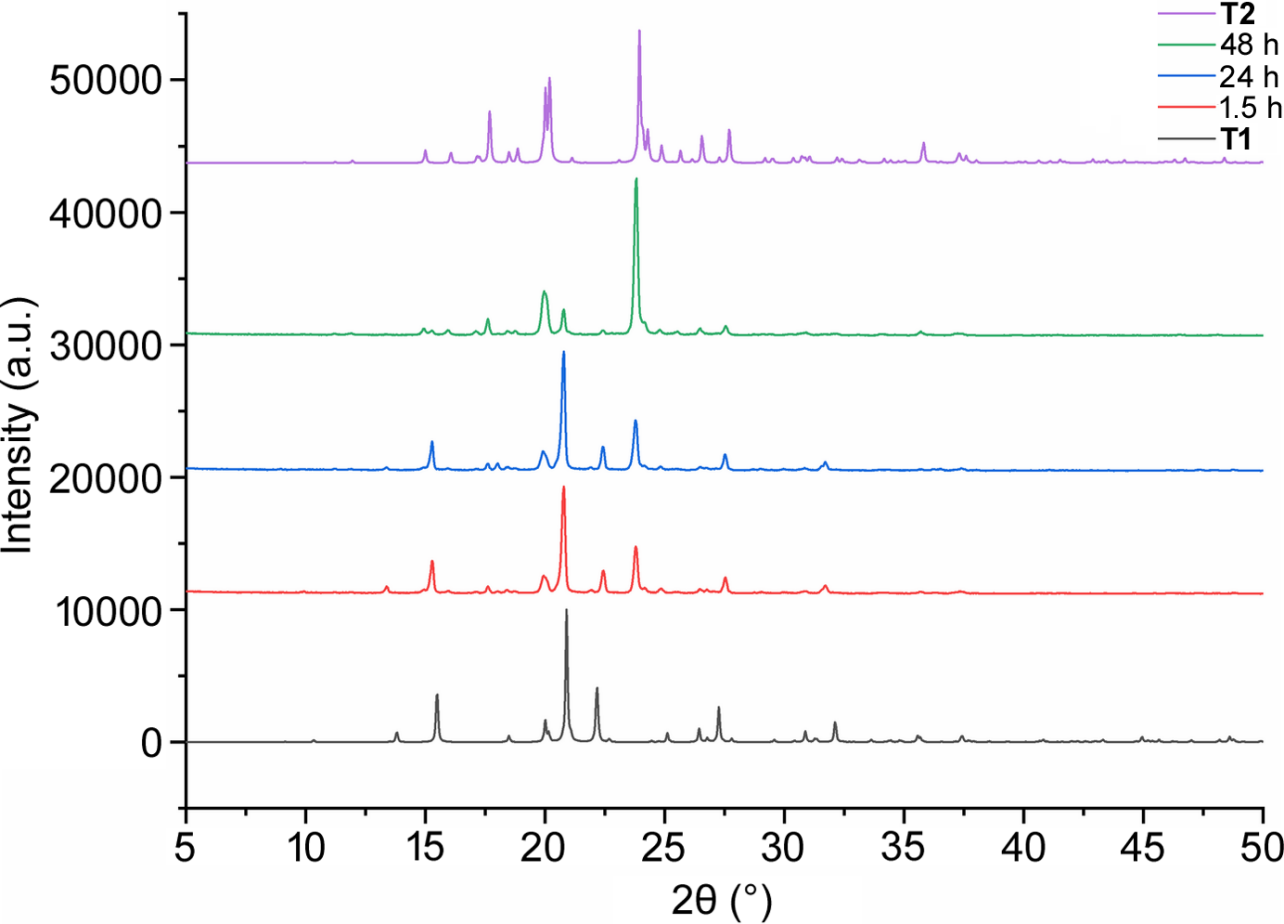
X-ray diffraction patterns showing the time evolution of the crystallization batch.

Powder X-ray diffraction (PXRD) analysis was conducted to assess the stability of the tyramine form (T1) obtained. The following methodology was employed: five identical syntheses were carried out simultaneously. After specified time intervals (1.5, 24 and 48 h), the products were filtered using a Büchner funnel, washed with a small amount of aceto­nitrile and left to dry. The dried products were then ground and analysed. Fig. 2 ▸ shows a comparison of the recorded diffraction patterns with those for pure T1 and T2 forms as obtained from simulated PXRD patterns from single-crystal X-ray diffraction (SCXRD) experiments.

The powder X-ray diffraction measurements were performed on an X’Pert PRO MPD diffractometer, equipped with a diffracted-beam graphite monochromator, a PIXcel1D detector and a Cu long fine focus ceramic tube. The diffraction data were collected at 298 K over the 2θ range from 3 to 80° with a step size of 0.02°.

### 2.4. Theoretical calculations

Using the *CRYSTAL17* software (Dovesi *et al.*, 2018 ▸), the experimental atomic positions in the tyramine polymorphs were optimized [the T2 geometry was taken from CSD entry SENJEC (Quevedo *et al.*, 2012 ▸)]. The wavefunctions generated for these optimized geometries were used to determine the theoretical electron density. The lattice parameters were fixed at the experimental values in order to retain maximum similarity of the optimized structures with the experimental ones. The *TOPOND14* software (Gatti & Casassa, 2017 ▸) was utilized for the topological analysis of electron density within the QTAIM (Bader, 1990 ▸) framework.

Optimized structures were used for further intermolecular lattice energy and total bulk energy calculations. The intermolecular lattice energy was computed using equation (1):
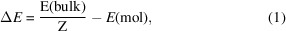
where *E*(bulk) is the total energy of the unit cell and must be referred to the number, *Z*, of molecules in the unit cell, and *E*(mol) is the total energy of the isolated molecule in the gas phase. Computed data were corrected for the BSSE through the counterpoise method. All calculations were performed using the B3LYP functional (Lee *et al.*, 1988 ▸; Becke, 1993 ▸) 6–311++G(d,p) basis set combined with an empirical dispersion energy correction (Civalleri *et al.*, 2008 ▸) implemented in the *CRYSTAL17* program (Dovesi *et al.*, 2017 ▸, 2018 ▸).

To obtain the initial lattice dynamical model for normal-mode refinement, periodic DFT vibrational frequencies at the Γ point were computed at the same level of theory, within the harmonic approximation, by diagonalizing the mass-weighted Hessian matrix. The vibrational modes were analysed and then the first six were refined using normal-mode refinement via the NoMoRe server (https://.nomore.chem.uw.edu.pl; Hoser *et al.*, 2021 ▸).

### 2.5. Differential scanning calorimetry

Differential scanning calorimetry measurements were performed for crystals of T1 and T2 using a DSC822e calorimeter. The samples were placed in an aluminium crucible (T1 – 8.45 mg; T2 – 3.35 mg) and first cooled down from room temperature (RT) to −140°C, then heated to 150°C and again cooled down to RT. All temperature changes were carried out at rate of 5°C min^−1^.

### 2.6. Raman spectra

Raman microscopy measurements were performed using a Renishaw Qontor inVia spectrometer equipped with a Leica microscope (50× long-range objective lens, NA = 0.5). The samples were excited using a 532 nm wavelength diode-pumped solid-state laser. Spectra were obtained from several different spatial positions of the sample at 100 and at 300 K (RT). The spectral range was set between 3200 and 100 cm^−1^, with laser power maintained between 1–3 mW to avoid any damage or alteration to the sample. Spectral data were processed using the Renishaw *WiRE* software (versions 5.5 and 3.4) as well as *Omnic* (version 7.3). Band positions, widths and intensities were calculated using the curve-fitting procedures within the *WiRE* software.

### 2.7. Hot-stage microscopy

The Linkam LTS420 (D) hot stage, equipped with a PT100 platinum sensor providing >0.01°C resolution and mounted on a Zeiss Axio ScopeA1 microscope, was used for observation of the T1 and T2 crystals from RT up to 165°C (the literature melting point of tyramine). Observations were repeated on nine different samples over an extended period of time proving the reproducibility of the observed processes and validating the temperatures at which we observe the changes.

### 2.8. Optical properties calculations

Refractive indices and second-order nonlinear electric susceptibility tensors were calculated for T1 and T2 using the modified local field theory (QLFT) approach (Munn, 1980 ▸; Bounds & Munn, 1981 ▸; Hurst & Munn, 1986 ▸; Seidler *et al.*, 2016 ▸). The molecular properties were determined using MP2/6–311++G(d,p), with frequency dispersion effects introduced via B3LYP/6–311++G(d,p) static and dynamic polarizability tensors, following the method of Seidler & Champagne (2016 ▸). Molecular polarizability calculations were performed using *GAUSSIAN09* (Frisch *et al.*, 2009 ▸). The atomic contributions to the molecular polarizabilities were determined through a partitioning scheme implemented in the *AIMAll* program.

## 3. Results and discussion

### 3.1. Crystal structures and Hirshfeld surfaces of the polymorphic forms of tyramine

The methods for obtaining two polymorphic forms – a new monoclinic form (*Pc*, T1) and the previously known triclinic form (*P*1, T2) – along with two new multicomponent products are illustrated in Fig. 1 ▸. Both microwave radiation and the addition of barbital were utilized during the crystallization process. Crystals of T1 were observed 1.5 h after the experiment was conducted. After a specific time in solution (as shown in Fig. 2 ▸), the T1 form gradually transformed into T2, as confirmed by powder diffraction patterns collected from the crystals extracted from the crystallization batches.

**Figure 3:**
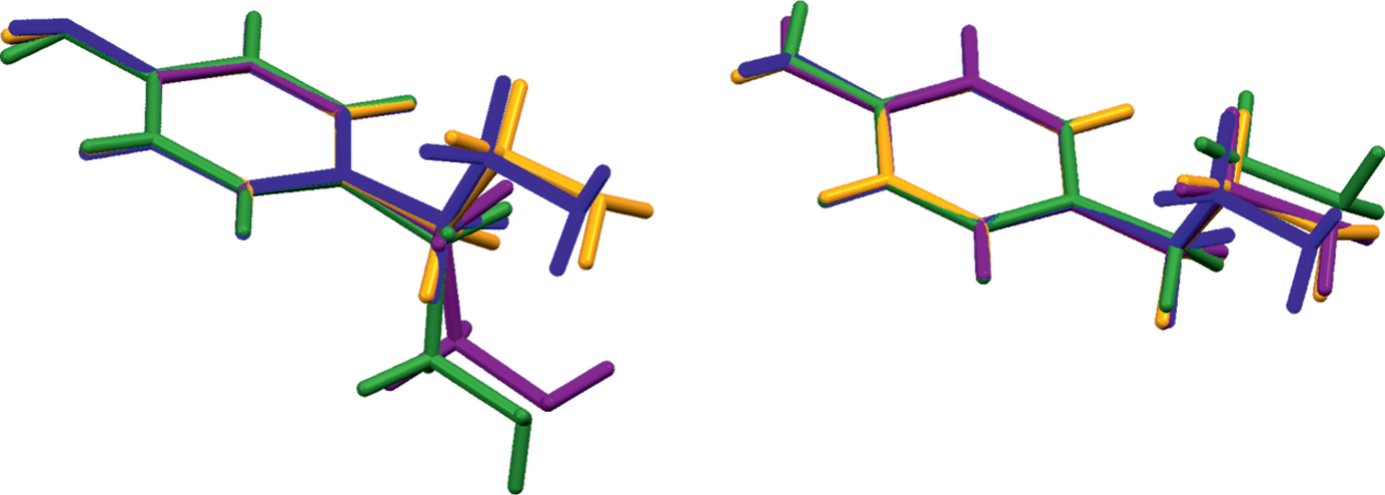
Overlay of tyramine molecules from polymorphic forms T1 and T2 in two different orientations. Orange and violet – T1; blue and green – T2.

The crystal structures of T1 and T2 were compared to identify the differences and similarities between these polymorphs. Both T1 and T2 contain two molecules in an asymmetric unit (*Z*′ = 2), as shown in Figs. S1 and S2 of the supporting information. At first glance, there are no significant differences in the alkyl chain conformation between them (Fig. 3 ▸); in all cases, the chain adopts an extended conformation, as confirmed by the torsion angles of the alkyl­amine chain (Table S1), indicating an antiperiplanar arrangement. However, in terms of conformation, the relative positions of the hydroxyl and amino groups are noteworthy. It is also important in this case because of the different orientations of the hydroxyl group (Fig. 3 ▸). Richardson *et al.* (2004 ▸) showed that, based on theoretical calculations, tyramine has seven conformers, only three of which feature an extended alkyl­amine chain (Richardson *et al.*, 2004 ▸). In T1, both molecules (Fig. S3) exhibit a conformation named C, which is the most stable among the extended forms. However, in T2 the D_2_ conformation is observed for both molecules, indicating a higher-energy state. The calculations were performed using a level of theory that has proven successful for similar molecules, with geometries that are consistent with the experimental data.

**Figure 4:**
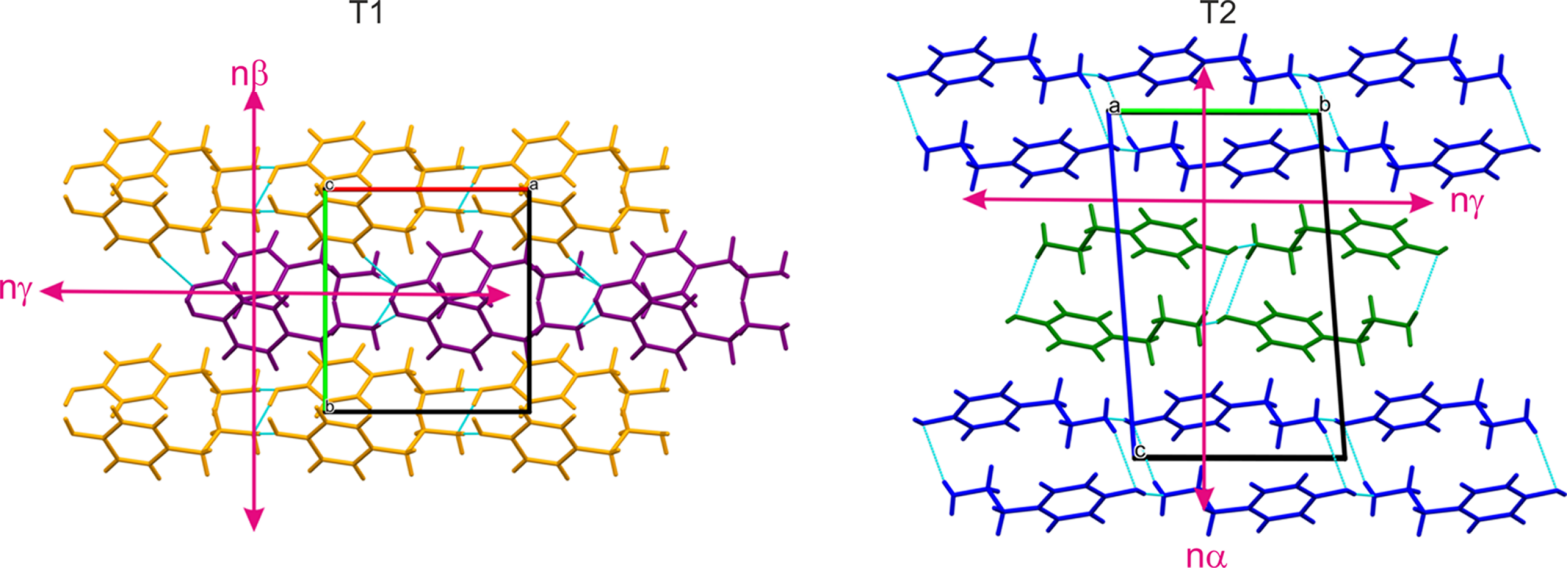
Packing of the structural components in T1 and T2 viewed in the [001] direction for T1 and [100] direction for T2. Different molecules from the asymmetric unit are presented in different colours.

An examination of the molecular packing in T1 and T2 (Fig. 4 ▸) reveals that both structures comprise two types of double chains, each formed by a distinct type of molecule. In T1 and T2, the shortest intermolecular interactions are similar in strength, with a slight advantage for T1, and they play a comparable role in the formation of the structures. An important aspect is the alignment of the chains: in T1, both types of molecules have the OH group pointing in one direction and the NH_2_ group located on the opposite side. In contrast, in T2, the molecules in the chains are placed in an antiparallel fashion. Based on this analysis it seems that the transformation from the T1 to T2 polymorphic form would require a rotation/reorganization of one strand of the double chain to create antiparallel systems. Also, in T2 the aromatic rings between the same types of molecules are exactly parallel, whereas in T1 they are close to perpendicular (87.44°, as shown in Fig. S5), but in fact this does not have a big impact on the intermolecular interactions (Figs. S6 and S7). C_ar_—H⋯O, C_aliph_—H⋯π and N—H⋯π are present in both polymorphs with similar distances between the hydrogen and acceptor. There are no π⋯π interactions in these structures. There are noticeable differences in the crystal density when comparing T1 (1.217 g cm^−3^) and T2 (1.274 g cm^−3^). Typically, there is a correlation between stability and crystal density: more stable polymorphs tend to exhibit higher packing densities as they minimize free energy more effectively. This suggests that T2 is the more stable phase. Ostwald’s Rule of Stages (Ostwald, 1897) similarly implies that during crystallization, when multiple metastable states are available, the system progresses step by step through the nearest thermodynamic state. However, this rule has exceptions. Recent studies (Cardew, 2023 ▸) have shown that, when classical nucleation theory is applied to polymorphic crystallization, metastable phases do not necessarily form sequentially but instead disappear in order of stability.

**Figure 5:**
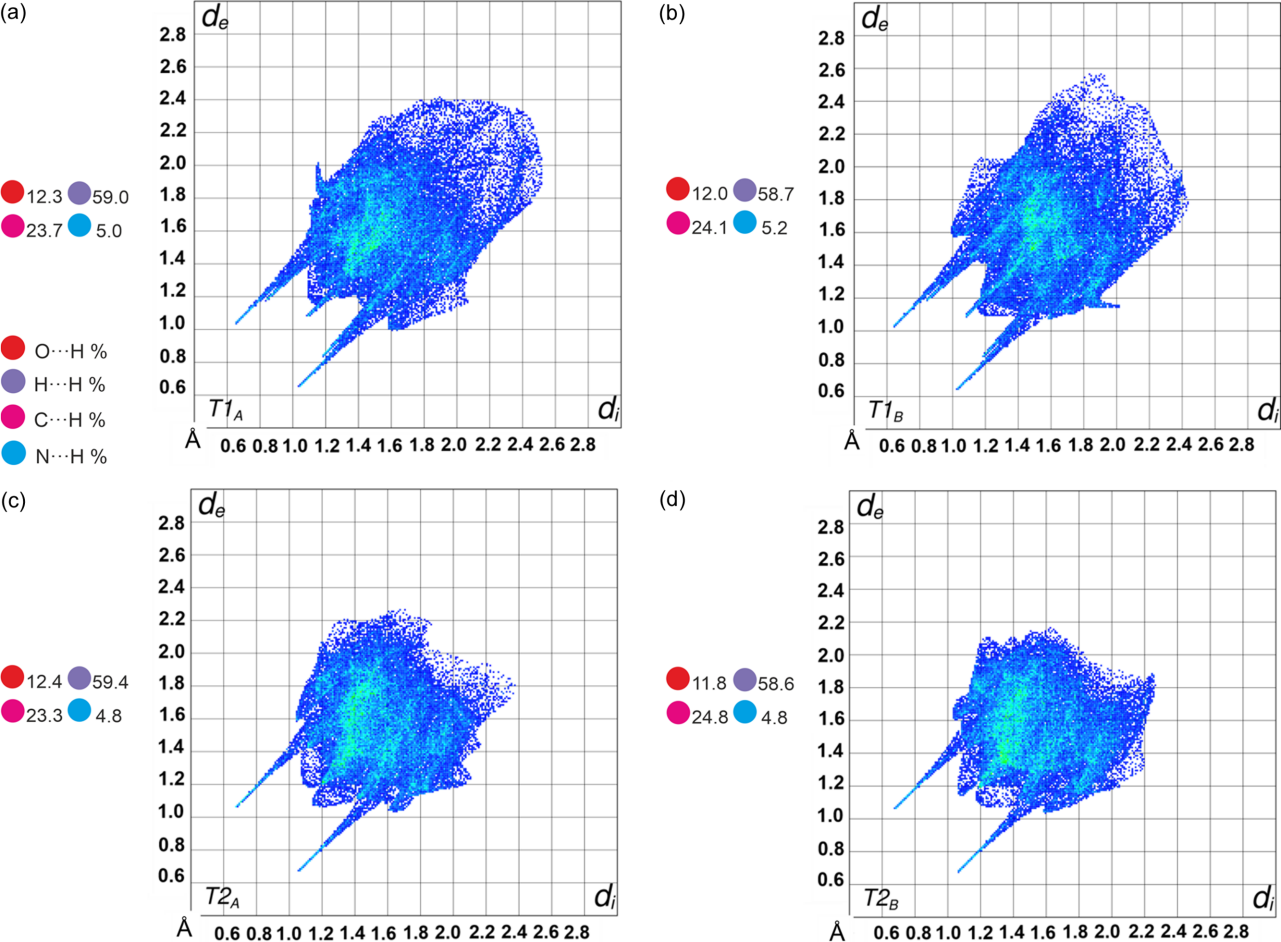
Fingerprint plots and percentage contributions of intermolecular contacts for molecules in T1 (top) and T2 (bottom). *d*_i_ and *d*_e_ represent distances from the Hirshfeld surface to the nearest atom inside and outside the surface, respectively.

Fingerprint plots (Fig. 5 ▸) were calculated from Hirshfeld surfaces using *Crystal Explorer* (Spackman *et al.*, 2021 ▸). Minimal differences in fingerprint shapes and the percentage contributions of each interaction indicate significant similarities in the interactions in T1 and T2. Characteristic spikes for low *d*_i_ and *d*_e_ values indicate N⋯H (O—H⋯N hydrogen bonds) interactions in all cases. Points on plots in the region of higher *d*_i_ and *d*_e_ (upper right corner) indicate C⋯H and H⋯H interactions, mostly C—H⋯π. Differences are connected only with distances in this type of interaction, which can be seen also from the density; a higher value for T2 indicates closer interatomic contacts.

### 3.2. Stability studies: hot-stage microscopy, DSC, Raman spectroscopy

**Figure 6:**
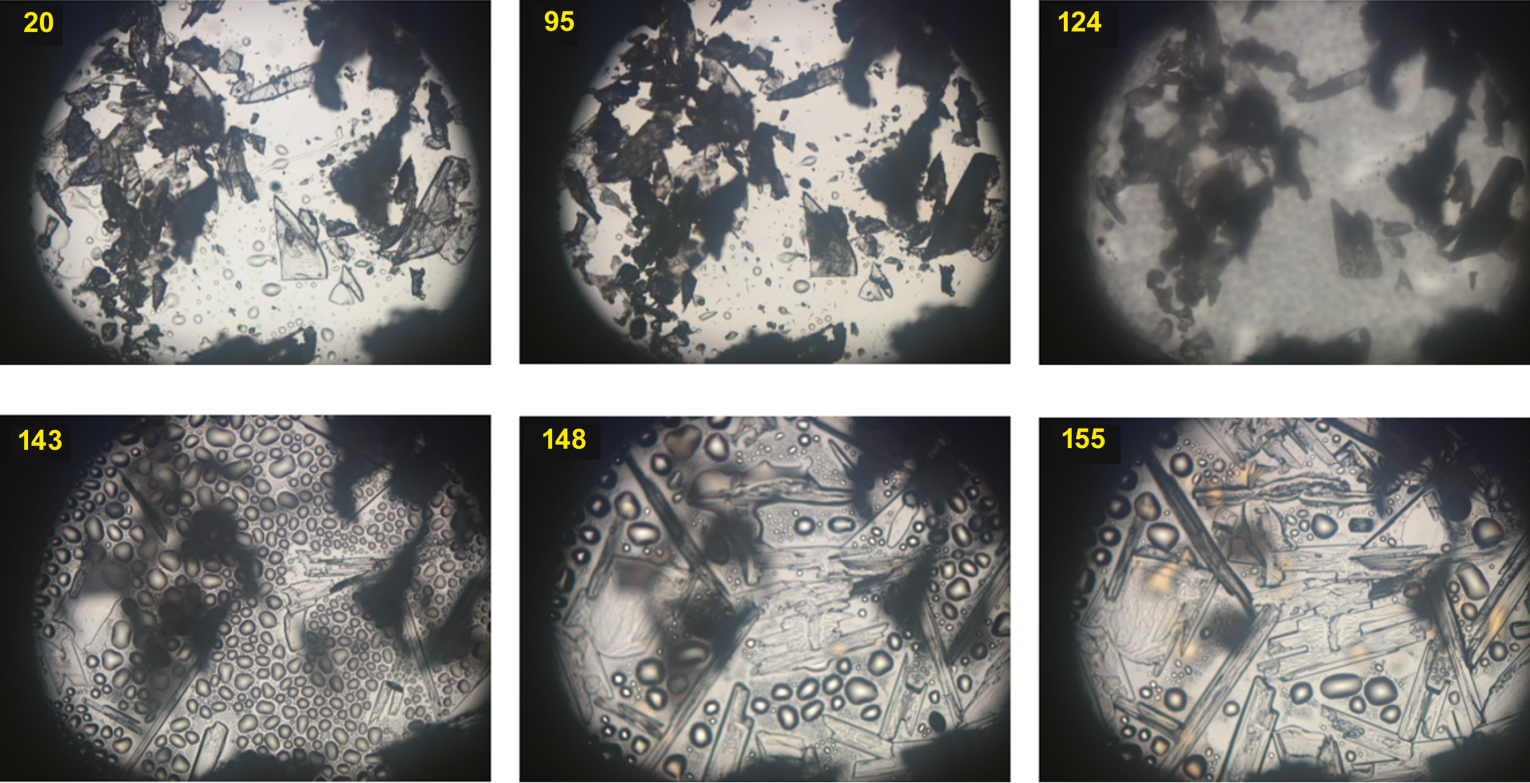
Hot-stage microscopic images of the tyramine T1 transformation to T2. Magnification 50×, temperature is marked in yellow (°C).

Hot-stage microscopy was employed to determine the thermal stability of the samples and to detect potential melting or decomposition points. Single crystals of T1 gradually became opaque on heating between 80 and 100°C, and completely melted at 140°C (Fig. 6 ▸). New crystals first appeared at 143°C and, at 154°C, we observed only the recrystallized form T2, confirmed by X-ray diffraction experiments. T2 melted at 163°C. We also examined the salt C2 using the same strategy, observing the crystals becoming opaque at 100°C. Melting began at 117°C and, at 135°C, we observed the recrystallization of a barbital polymorph (trigonal), as confirmed by X-ray diffraction experiments.

**Figure 7:**
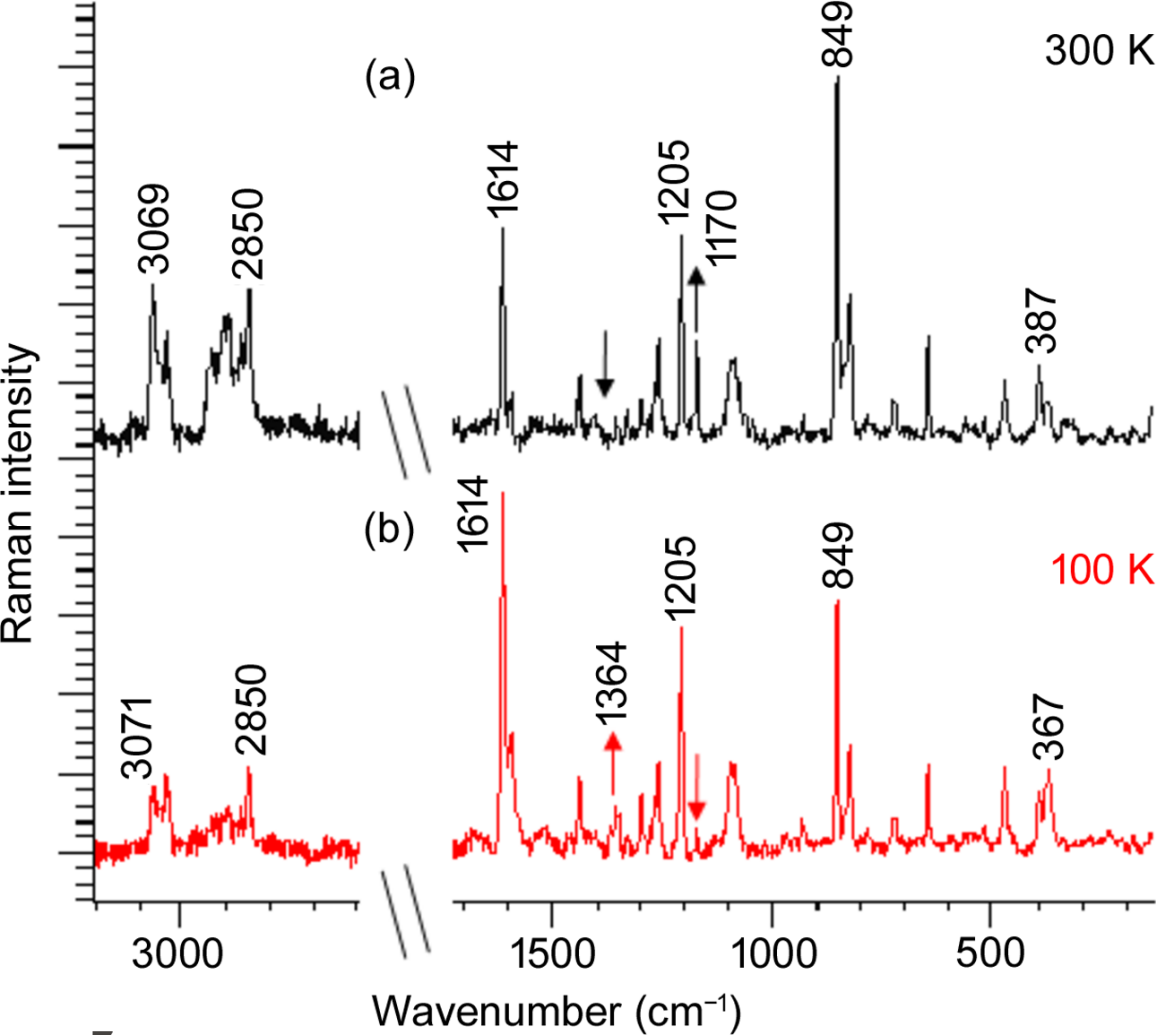
Averaged Raman spectra collected at (*a*) 300 K and (*b*) 100 K with a 532 nm laser line.

DSC analysis (Fig. S8) of the new polymorphic form T1 revealed distinct endothermic and exothermic peaks around −50°C. There are no similar changes in form T2 under the influence of temperature – this polymorph remains stable throughout the entire analysed range. To determine if T1 undergoes a phase transition at low temperatures, single-crystal X-ray diffraction measurements were conducted across a temperature range from room temperature (RT) to 100 K. The crystal structure of T1 remained unchanged on cooling. To further understand the DSC behaviour of T1, we analysed Raman spectra collected at both RT and low temperatures (Fig. 7 ▸ and Table S5). At first glance, the collected data do not reveal significant differences; however, there are variations in a few spectral regions. In the skeletal vibrations range 400–300 cm^−1^, the 367 cm^−1^ band, associated with CC vibrations adjacent to N, becomes more prominent at low temperatures, while the 397 cm^−1^ band, related to ring vibrations, is more intense at RT (Siddiqui *et al.*, 2009 ▸). Another distinction can be found by looking at the bands belonging to the doublet around 830 and 849 cm^−1^, associated with the Fermi resonance between the ring-breathing vibration and the ring-bending vibration of the para-substituted benzenes. The Raman spectrum at room temperature is characterized by the most intense band at 849 cm^−1^ (Tu, 1982 ▸). The band around 849 cm^−1^ dominates, which means that tyramine forms an ordered molecular structure.

On the other hand, in the low-temperature spectrum the band at 1614 cm^−1^ dominates, which indicates that the ring has complete freedom to perform breathing vibrations (see Fig.7). A significant enhancement of the intensity of symmetric vibrations associated with the ring may indicate the occurrence of interactions between aromatic rings in successive chains (Webster *et al.*, 2009 ▸). This vibration, with the geometry of this vibrating ring system, is also associated with the enhancement of the intensity of the CH vibrations at the 3040 and 3071 cm^−1^ positions. The C—H stretching vibration region is very sensitive to structural changes observed in aliphatic chains. The band at approximately 2090 cm^−1^ can be attributed to Fermi resonance between CH units on rings adjacent to hydro­carbon chains. Thus, the intensity of the 2900 cm^−1^ band is significant when the lateral packing between the chains becomes stable, as observed at RT. Intermolecular interactions can therefore occur at low temperatures when the intensity of this band decreases due to disruption of the lateral packing between the chains (Table S5). It has been found that not all —CH_2_ residues are equivalent, especially the CH_2_ residue adjacent to the NH_2_ terminus at 2940 cm^−1^, see Table S6. The 1170 cm^−1^ band, associated with ν(CC) vibrations adjacent to the ring and δ(CCC) bending vibrations, is particularly notable as it appears clearly at room temperature (marked by an arrow in Fig. 7 ▸). However, the 1364 cm^−1^ band is clearly observed at low temperature due to ν(CC) ring stretching and δ(HOC) deformation vibrations (marked by an arrow in Fig. 7 ▸). Temperature allows differentiation between the vibrational modes: ring vibrations are more pronounced at 300 K, while vibrations in the chain region near the N atom exhibit greater intensity at 100 K. The data suggest that temperature plays a significant role in influencing molecular vibrations and interactions, particularly those involving aromatic rings in T1. This is indicated by the previously mentioned region of CH stretching vibrations typical for aromatic systems, where the bands at 3040 and about 3071 cm^−1^ are observed, with almost identical intensities for 100 K and a more intense component at about 3071 cm^−1^ for crystals at 300 K (see Table S5). These findings imply that while the crystal structure remains stable (assuming an averaged model in time and space), the molecular dynamics within T1 are sensitive to temperature changes. Overall, the data indicate that polymorph T1, despite having a stable crystal structure under cooling, exhibits temperature-sensitive vibrational modes and molecular interactions that are not present in the more thermally stable polymorph T2. These differences in thermal behaviour and molecular dynamics between T1 and T2 provide insights into their potential applications and stability under varying conditions.

### 3.3. Theoretical charge density studies and formation energies evaluation of T1 and T2

For T1 and T2 theoretical charge density analysis using optimized crystal structures was performed to understand the nature and emphasize the differences between the polymorphs. Due to the low quality of crystals obtained from numerous crystallization trials, it was not possible to determine the experimental charge density distribution. The electron density, Laplacian, local energy densities and approximated interaction energy evaluated at the bond critical points (BCPs) for the strongest intermolecular interactions are shown in Table 1 ▸. Their choice was dictated by the highest value of the energy and ρ(**r**) at the BCP.

In both T1 and T2, the strongest interactions can be classified as intermediate between closed-shell and shared-shell, *e.g.* based on |*V*(**r**_CP_)|/*G*(**r**_CP_) values which are higher than 1. It is also confirmed by the values of the total energy densities evaluated at the BCP, which are visibly lower than zero. In both cases, these interactions are hydrogen bonds building the same synthon: OH⋯NH_2_, which created the chains mentioned before. The above-mentioned hydrogen bonds in T1 and T2 show the highest energies (approximately −20 kcal mol^−1^) with T1 having an advantage of about −4 kcal mol^−1^. The last hydrogen bond, H12⋯O1A, is weaker relative to the remaining ones. Similar hydrogen bonds in T2 (H2B1⋯O1B and H2A2⋯O1A) have lower energies than H22⋯O1B in T1 and higher than H12⋯O1A, but overall they are comparably weaker. This analysis shows that the most important hydrogen bonds are in total slightly stronger for the polymorph T1. The observed differences in thermal behaviour between T1 and T2 correlate with their thermodynamic properties, which are governed by the Gibbs free energy. The form with the lower Gibbs free energy under given conditions (such as temperature and pressure) is more stable at those conditions. To accurately calculate the Gibbs free energy, one must consider not only the contributions from the electronic energy, which correspond to enthalpy, but also those arising from vibrations such as zero-point energy (ZPE), entropy (*S*) and vibrational enthalpy (*H*_vib_) for both polymorphic forms under study. To calculate vibrational contributions, the vibrational frequencies of the crystal are necessary. Moreover, to obtain accurate values, frequencies should be calculated not only at the Γ point of the Brillouin zone (BZ) but also at other points, meaning the BZ should be carefully sampled. These calculations are computationally expensive. Therefore, we decided to apply the dynamic quantum crystallography approach, in which frequencies obtained at the Γ point are refined using single-crystal X-ray diffraction data (Hoser *et al.*, 2016 ▸; Hoser *et al.* 2017 ▸; Hoser *et al.*, 2021 ▸). For both T1 and T2, we conducted normal-mode refinement and for six refined frequencies we obtained a model with low *wR*2 and reasonable ADPs. The thermodynamic properties obtained are shown in Table 2 ▸.

The intermolecular lattice energies calculated for polymorphs T1 and T2 are similar (see Table S9 in the supporting information for more details). However, their total energies differ — T2 exhibits an energy that is lower by 2 kJ mol^−1^ compared with T1, making T2 the more stable form at low temperatures. According to theoretical DFT frequency calculations at the Γ point, form T2 is further stabilized by entropy. After conducting normal-mode refinement, the frequencies obtained were used to calculate entropy, revealing that T1 has a slightly higher vibrational entropy (0.1 kJ mol^−1^). This result aligns with expectations, as the larger unit cell and lower density of T1 provide more space for atomic and molecular vibrations. However, the entropy difference between T1 and T2 is extremely small and within the methodological limits. This minor difference is insufficient to overcome the electronic energy advantage of T2 or reverse the stability order of T1 and T2 before T1 melts. Thus, T1 and T2 form a monotropic system, with T2 being the more stable polymorph.

All these findings align with experimental observations. T1 melts at a significantly lower temperature, and near the melting point T2 is the more stable form. There is no solid-to-solid phase transition; T1 melts first, after which T2 crystallizes from the melt. At low temperatures, the primary difference between T1 and T2 lies in the energy of their molecular conformations – molecules in T2 adopt more energetically favourable conformations. We can hypothesize that the use of unconventional microwave-assisted crystallization enables access to alternative molecular conformations and, consequently, to a metastable polymorph.

### 3.4. Crystal structures of C1 and C2

**Figure 8:**
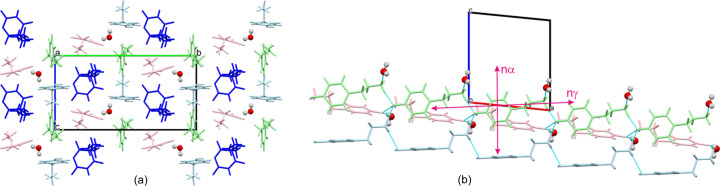
(*a*) Packing of the structural components in C1 viewed along the [100] direction. (*b*) Triple strand of chains. Cation B is highlighted in blue, zwitterions C and D are in green and pink, respectively, and barbital anions are highlighted in navy blue.

As shown in Fig. 1 ▸ and described in Section 2.1, the salt and salt co-crystal were obtained in the same crystallization batch after an appropriate period of time and/or the solvent evaporation. The salt co-crystal C1 crystallizes in the monoclinic space group *P*2_1_. The asymmetric unit (Fig. S11) contains one barbitalate anion (labelled A), one tyraminium cation (labelled B), two tyraminium zwitterions (labelled C and D) and one water molecule. The crystal structure of C2 follows the symmetry of the *R*3*c* space group. The unit cell is characterized by a very long *c* period (62.5609 Å). The asymmetric unit of C2 contains one barbital anion (labelled A), one tyraminium cation (labelled B) and two partially occupied aceto­nitrile molecules disordered across a symmetry element (Fig. S12, see Table S1 for the chemical composition). The C1 structure represents the first known instance where tyramine molecules exist as zwitterions. A search of the Cambridge Structural Database (as of August 2024) revealed no other organic crystal structures containing tyramine with this characteristic. Both zwitterions are connected to each other via a water molecule (O—H⋯O and N—H⋯O hydrogen bonds). The three tyramine moieties form a triple-strand chain motif along the [100] direction. Each of the strands is interconnected through hydrogen bonds involving barbital anions and water molecules (Fig. 8 ▸).

In both structures studied there are rare barbitalate ions formed through deprotonation of the barbital N3 atom. There are fourteen records in the CSD containing the barbitalate anion and only six of them were formed by proton transfer to the amino group.

**Figure 9:**
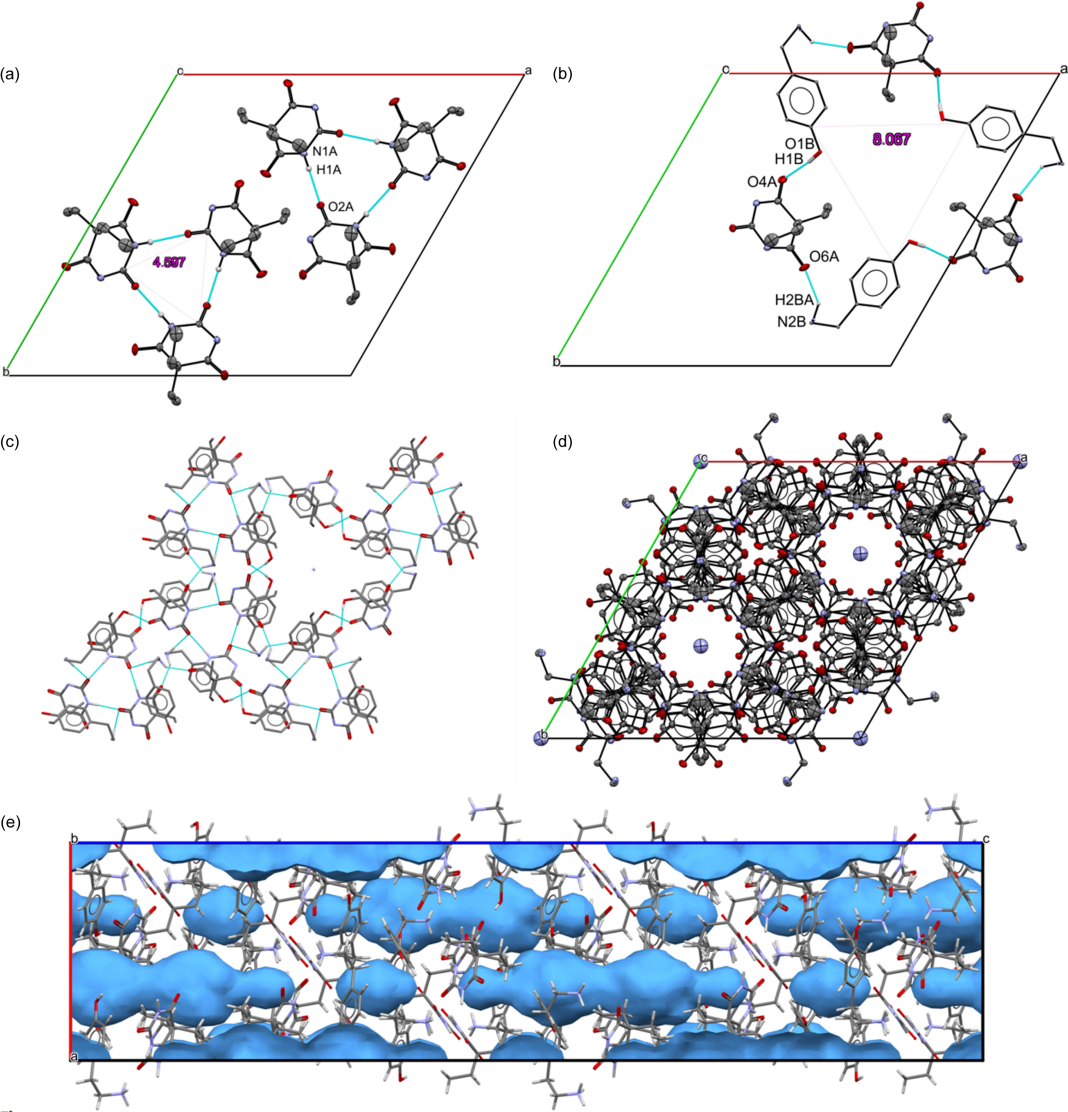
(*a*) Two 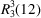 ring motifs at *z* = 0.1–0.3. (*b*) 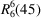 ring motif at *z* ≃ 0.2. (*c*) Single layer in C2 at *z* = 0 – 1/6. (*d*) Crystal packing of C2 components with solvent molecules, viewed along [001]. Hydrogen atoms have been omitted for clarity. (*e*) Side view of the channels, with the voids marked in blue. Visualization after the SQUEEZE procedure.

**Figure 10:**
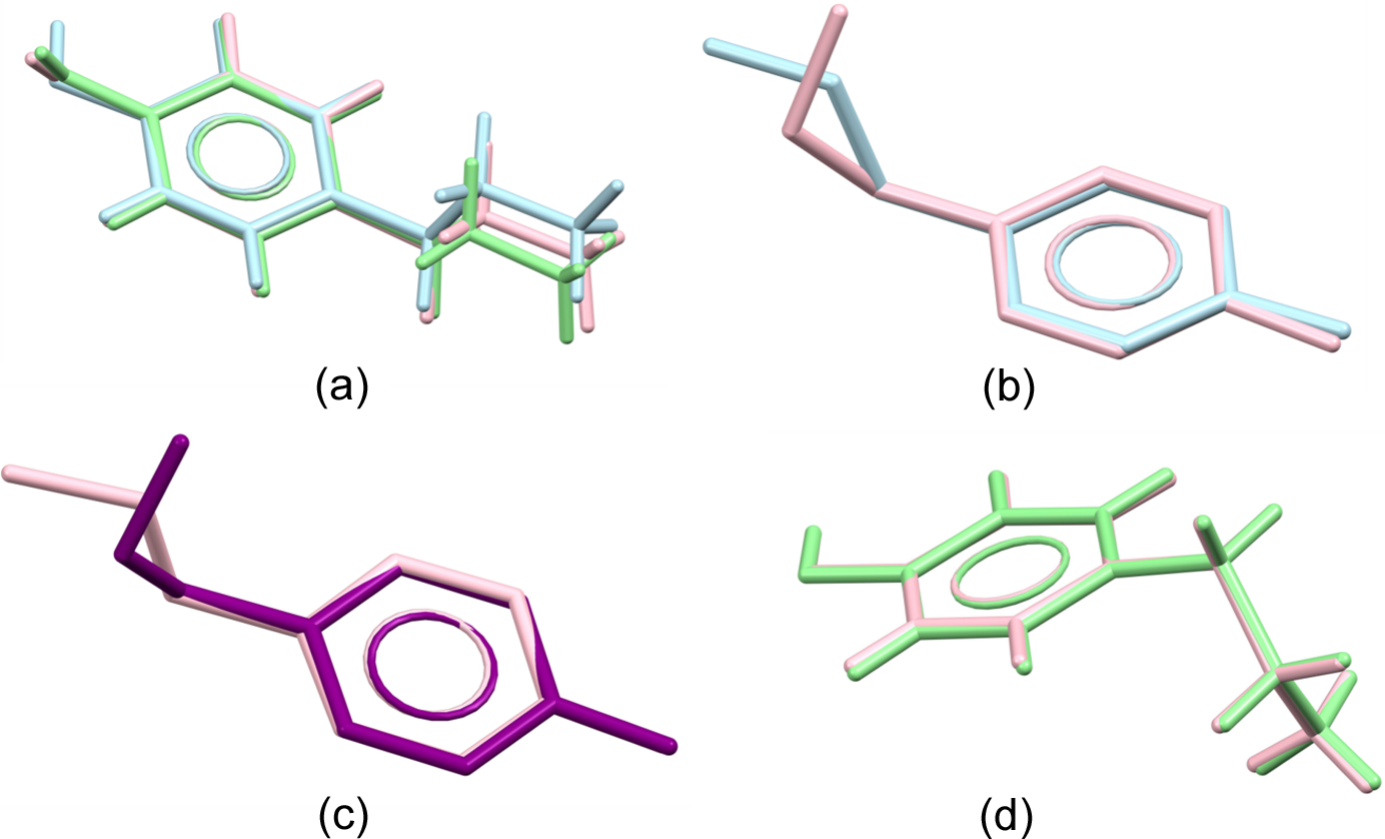
(*a*) Overlay of the tyraminium cation and two tyraminium zwitterions in C1, showing cation B in blue, zwitterion C in green and zwitterion D in pink. (*b*) Overlay of the tyraminium cations taken from studied polymorphs C1 (pink) and C2 (blue). (*c*) Overlay of the tyramine zwitterion taken from studied polymorph C1 (purple) and tyraminium cation from C2 (pink). (*d*) Overlay of the tyraminium cations taken from studied pseudopolymorph C2 (pink) and (II) (green). Hydrogen atoms have been omitted for clarity.

The C2 structure is an isomorph of the tyraminium 5,5-di­ethyl­barbiturate ethanol/water solvate obtained in our previous research (Rydz *et al.*, 2018 ▸). In both structures the barbitalate ions form 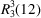 rings [Fig. 9 ▸(*a*)] via intermolecular N1A—H1A⋯O2A^i^ hydrogen bonds forming trimers. Tyraminium cations and barbitalate anions are arranged in large 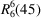 motifs through intermolecular N2B—H2BA⋯O6A^ii^ and O1B—H1B⋯O4A^iii^ hydrogen bonds [Figs. 9 ▸(*b*), 9 ▸(*c*) and 9 ▸(*d*)]. The crystal structure of C2 is composed of layers, each with a thickness of 1/6*c*. Within each layer, two small barbitalate motifs and one large ring motif are present, stabilized by tyraminium cations. Solvent molecules (aceto­nitrile) are located in cavities parallel to the [001] direction [Fig. 9 ▸(*e*)]. The voids [Fig. 9 ▸(*e*)] in C2 (probe radius = 1.2 Å and grid spacing = 0.7 Å) occupy 10.7% of the unit-cell volume (calculated in *Mercury*), which corresponds to 1722.48 Å^3^. The aceto­nitrile molecules do not participate in the formation of hydrogen bonds with the surrounding barbitalate and tyramine ions. Details of the hydrogen-bond geometry for C1 and C2 are given in Tables S7 and S8. The analysis of the tyramine ion and zwitterion conformation in C1 revealed similar torsion angles of the side chain C5—C8—C9—N1 (in the range 175–179°). However, the cations in C1 and C2 adopt different conformations. In C1, the side chain is extended, whereas in C2, it folds toward the aromatic ring (C5—C8—C9—N1 torsion angle of 64.5°). A visual comparison of the tyraminium cation and zwitterion conformations in both C1 and C2 structures is shown in Fig. 10 ▸. These conformational differences influence the crystal packing due to the varying hydrogen-bonding tendencies of the tyraminium cations. In C1, the extended side chain of the cation promotes the formation of a hydrogen-bonded chain motif through the N1B—H1NB⋯O1B^iv^ hydrogen bond, a preference also observed for the tyramine zwitterions in C1. In contrast, in C2, the folded side chain forms 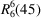 motifs through N2B—H2BA⋯O6A^ii^ and O1B—H1B⋯O4A^iii^ hydrogen bonds. These differences are further illustrated in the fingerprint plots of the tyraminium cations and zwitterions presented in the next section.

### 3.5. Hirshfeld surfaces analysis for C1 and C2

**Figure 11:**
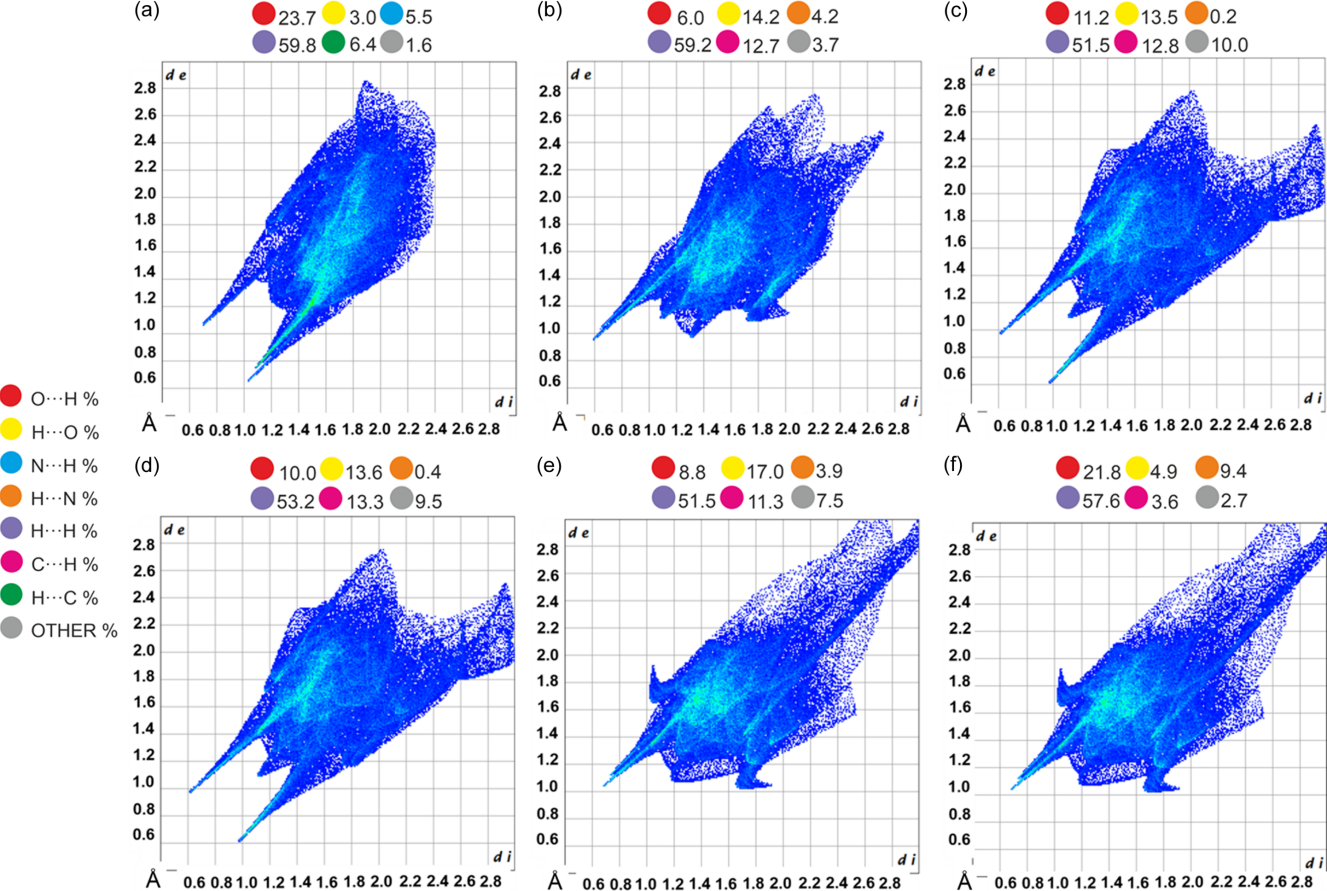
Fingerprints plots for the (*a*) barbitalate anion, (*b*) tyraminium cation, (*c*) tyraminium zwitterion C and (*d*) tyramine zwitterion D in the crystal structure of C1; (*e*) the tyraminium cation and (*f*) the barbitalate anion in the crystal structure of C2. The dots above the plots show the percentage contribution of the intermolecular interactions. Visualization after applying the SQUEEZE procedure.

The results of the Hirshfeld surface (HS) and fingerprint plot analyses for all ions in C1 are shown in Fig. 11 ▸. HS analysis reveals that the C1 structure is primarily stabilized by weak H⋯H interactions, represented by diffuse points in the middle of the plot. The percentage contributions of H_in_⋯O_out_ and O_in_⋯H_out_ interactions highlight significant differences among the ionic species, where ‘in’ subscript represents an atom inside the surface, whereas ‘out’ denotes the atom outside the surface. The barbitalate anions exhibit nearly four times as many O_in_⋯H_out_ interactions compared with the tyraminium cation, and over twice as many compared with the tyramine zwitterions. Conversely, both the tyraminium cation and the zwitterions show greater involvement in forming H_in_⋯O_out_ interactions. The differences observed in interaction patterns suggest that the barbitalate anions are more engaged in accepting hydrogen bonds. On the other hand, the tyraminium cation and zwitterions seem to play a complementary role as good donors, helping to build the hydrogen-bonding network that supports the crystal architecture.

As can be seen in the fingerprint plots for C2 (Fig. 11 ▸), the H⋯H contacts represent for more than 50% of all interactions. The major difference between the tyraminium cation and barbitalate anion is the participation of these ions in the formation of H_in_⋯O_out_ and O_in_⋯H_out_ interactions. The percentage contribution of H_in_⋯O_out_ and O_in_⋯H_out_ for the tyraminium cation and barbitalate anion are 17.0 and 8.8%, and 9.4 and 21.8%, respectively. The contribution of the remaining interaction is less than 10%. Comparison of the distribution of O_in_⋯H_out_ interactions of the tyraminium cations in the C1 and C2 shows that these interactions are of similar strength (short *d*_e_ and *d*_i_). However, the H_in_⋯O_out_ contacts are weaker in the case of C2. The number of H⋯H contacts is about 8% less in C2 (51.5%) compared with C1 (59.1%).

### 3.6. Optical properties

Given that the crystal structure of T1 adopts the noncentrosymmetric, polar space group *Pc*, with all tyramine molecules aligned in the same direction, we explored their (non)linear optical properties in greater detail. The static and dynamic refractive indices, as well as the χ(2) tensor components calculated using the QLFT methodology, are presented in Table 3 ▸. Assuming Kleinman symmetry, six non-zero susceptibility tensor components were identified. The investigated T1 crystals demonstrated a moderate second harmonic generation (SHG) effect, with the maximum χ(2) component around 5 pm V^−1^. This result, while expected given that tyramine is not a push–pull system, suggests that mimicking the crystal structure of T1 with a molecule possessing higher molecular hyperpolarizability could yield substantial optical effects. Phase-matching configurations for type I and II interactions were derived based on the theoretically predicted dispersion of the refractive indices for both T1 and T2 (Fig. S9). The effective nonlinear coefficient, *d*_eff_, was mapped in these directions (Fig. S10). Unfortunately, pure bulk-phase T1 was not obtained in sufficient quality for SHG measurements via the Kurtz–Perry method. Both T_1_ and T_2_ crystals are biaxial positive and display notable linear birefringence, with Δ*n*_T_1__ = 0.164 and Δ*n*_T_2__ = 0.255, respectively (for reference, calcite, renowned for its significant birefringence, exhibits Δ*n* = 0.172). Optical indicatrix analysis revealed that the semiaxis corresponding to the highest refractive index, *n*γ, aligns closely with the direction of the tyramine double chains (see Fig. 4 ▸). In contrast C1 crystals are also biaxial positive, but exhibit significantly lower birefringence (0.095), indicating reduced optical anisotropy. In this case the variation in refractive indices along different crystallographic directions is less pronounced compared with T_1_ and T_2_. Similarly to T1 and T2 the highest refractive index can be found along the triple chain motif (Fig. 8 ▸). This trend of more evenly distributed values and reduced optical anisotropy is also reflected in second-order susceptibility tensor components (Table 3 ▸).

## 4. Conclusions

In this study, we examined the thermal behaviour, optical properties and molecular dynamics in two polymorphic forms of tyramine, T1 and T2, using quantum crystallography and Raman spectroscopy. Polymorph stability is determined by many factors, including the overall balance of intermolecular forces, packing efficiency and lattice energy, not just the strength of individual hydrogen bonds. In the case of T1 and T2, although T1 has slightly stronger hydrogen bonds, T2 exhibits a more favourable overall balance of these factors, contributing to its higher stability. Noticeable differences in crystal density between T1 (1.217 g cm^−3^) and T2 (1.274 g cm^−3^) suggest that T2 is the more stable phase, as higher density often correlates with greater stability. While Ostwald’s Rule of Stages (1897) suggests sequential progression through metastable states during crystallization, recent studies (Cardew, 2023 ▸) indicate that, in polymorphic systems, metastable phases may disappear in order of stability rather than forming sequentially. Our system exemplifies a situation where T1 forms first, in line with Ostwald’s Rule, and then disappears in favour of T2, which reflects the more recent theory of metastable phase disappearance.

In addition to the polymorphs T1 and T2, we explored the evolution of the system when barbital was introduced as an additive. Initially, T1 crystallizes under microwave radiation in the presence of barbital, transforming into T2 after a period of time. On leaving the crystals in solution, the C1 form emerges. The structure of C1 is particularly noteworthy as it represents the first known instance of tyramine molecules existing as zwitterions. The C1 structure retains some features reminiscent of the chain motifs found in T1 and T2, with the three tyramine moieties forming a triple-strand chain motif interconnected through hydrogen bonds involving barbital anions and water molecules. When the solution is left for a longer period, the C2 form develops. This structure is characterized by a different conformation of tyramine, where the side chain folds toward the aromatic ring. Unlike C1, the C2 form is a true salt, and the conformation of the tyraminium cation in C2 differs significantly from that in C1, influencing the hydrogen-bonding patterns and overall crystal packing. C1 also exhibits biaxial positive behaviour, similar to T1 and T2, but with significantly lower birefringence (Δ*n* = 0.095), indicating reduced optical anisotropy. This corresponds to the smaller optical anisotropy observed in those crystals. Despite the lower birefringence, the highest refractive index in C1 is still aligned with the triple-chain motif, showing a structural consistency with the T1 and T2 forms.

In summary, our study highlights the complex interplay between polymorphism and salt formation in the tyramine–barbital system. The evolution from T1 to C1 and eventually to C2 reflects the delicate balance of factors such as intermolecular interactions, solvent effects and crystallization conditions, all of which contribute to the stability and structural diversity observed in these materials. Polymorph T2 remains the most stable under the conditions studied, while the emergence of C1 and C2 illustrates the dynamic nature of molecular assembly processes in the presence of additives like barbital. These findings offer important insights into the design and control of polymorphic and salt forms in the context of pharmaceutical development and materials science, highlighting the impact of additives and crystallization conditions on structural outcomes.

## 5. Related literature

The following reference is cited in the supporting information: Sovago *et al.* (2020 ▸).

## Supplementary Material

Crystal structure: contains datablock(s) T1, T2, C1, C2. DOI: 10.1107/S2052252525002210/pen5003sup1.cif

Structure factors: contains datablock(s) T1. DOI: 10.1107/S2052252525002210/pen5003T1sup2.hkl

Structure factors: contains datablock(s) T2. DOI: 10.1107/S2052252525002210/pen5003T2sup3.hkl

Structure factors: contains datablock(s) C1. DOI: 10.1107/S2052252525002210/pen5003C1sup4.hkl

Structure factors: contains datablock(s) C2. DOI: 10.1107/S2052252525002210/pen5003C2sup5.hkl

Supporting tables and figures. DOI: 10.1107/S2052252525002210/pen5003sup6.pdf

CCDC references: 2380328, 2380329, 2380335, 2380336

## Figures and Tables

**Table 1:** Topological analysis of the chosen intermolecular interactions at the BCP (−3, 1) for T1 and T2

Interaction	ρ(**r**) (e Å^−3^)	∇^2^ρ(**r**) (e Å^−5^)	*G*(**r**_CP_) (e^2^ Å^−4^)	*V*(**r**_CP_) (e^2^ Å^−4^)	*E*(**r**_CP_) (e^2^ Å^−4^)	|*V*(**r**_CP_)|/*G*(**r**_CP_)	*G*(**r**_CP_)/ρ(**r**)	*E*(**r**_CP_)/ρ(**r**)	*E*_int_ (kcal mol^−1^)†
T1									
H1B⋯N2B	0.51	2.06	0.32	−0.50	−0.18	1.55	0.63	−0.35	−23.39
H1A⋯N2A	0.50	2.08	0.32	−0.49	−0.17	1.54	0.63	−0.34	−22.87
H22⋯O1B	0.14	2.07	0.13	−0.11	0.02	0.85	0.88	0.13	−5.03
H12 ⋯O1A	0.02	0.25	0.01	−0.01	0.00	0.71	0.69	0.20	−0.44

T2									
H1A⋯N2A	0.43	2.15	0.28	−0.41	−0.13	1.46	0.65	−0.30	−18.99
H1B⋯N2B	0.43	2.15	0.28	−0.41	−0.13	1.46	0.65	−0.30	−18.98
H2B1⋯O1B	0.04	0.63	0.03	−0.02	0.01	0.66	0.75	0.26	−1.00
H2A2⋯O1A	0.04	0.56	0.03	−0.02	0.01	0.70	0.71	0.21	−0.99

† Interaction energy approximated using Espinosa *et al.* (1998 ▸).

**Table 2:** Tyramine polymorphs and their thermodynamic properties All energies are reported per single molecule of tyramine. *E*_latt_ – intermolecular lattice energy, *E*_bulk_ – total energy, *S* – vibrational entropy estimate, *G* – Gibbs free energy. Relative differences in energy (Δ) were calculated by subtracting a given energy of the T1 form from that of the T2 form, *e.g.* Δ*E* for the T1 form is always 0.

	T1	T2
Crystal system	Monoclinic	Triclinic
Space group	*P*c	*P* 1
*Z*′	2	2
*V* (Å^3^)	748.8	714.9
Crystal density (g cm^−3^)	1.217	1.274
Melting point (°C)	140	163

Periodic DFT calculations		
*E*_latt_ (kJ mol^−1^)	−164.4	−164.3
Δ*E*_latt_ (kJ mol^−1^)	0	0.1
Δ*E*_tot_ (kJ mol^−1^)	0	−2.0

Vibrational contributions (298 K) from frequencies from Γ point of BZ		
*T*Δ*S* (kJ mol^−1^)	0	0.9
Δ*H*_vib_ + ΔZPE (kJ mol^−1^)	0	0.5
Δ*G* (kJ mol^−1^)	0	−2.4

Vibrational contributions (298 K) from frequencies from normal-mode refinement		
*T*Δ*S* (kJ mol^−1^)	0	−0.1
Δ*H*_vib_ + ΔZPE (kJ mol^−1^)	0	0.4
Δ*G* (kJ mol^−1^)	0	−1.5

**Table 3:** Results of QLFT MP2/6-311++G(d,p) calculations for T1 and T2; χ(2) tensor components (pm V^−1^)

Method	λ (nm)	*n_z_*	*n_y_*	*n_x_*	χ_111_	χ_113_	χ_311_	χ_122_	χ_212_	χ_133_	χ_313_	χ_223_	χ_322_	χ_333_
T1	∞	1.674	1.530	1.515	4.8	−0.1	−0.1	0.5	0.5	0.2	0.2	−0.2	−0.2	−0.5
	1064	1.683	1.539	1.524	5.2	−0.2	−0.2	0.6	0.6	0.2	0.2	−0.3	−0.3	−0.7
	532	1.716	1.567	1.552										

T2	∞	1.572	1.749	1.512										
	1064	1.581	1.761	1.520										
	532	1.610	1.801	1.546										

	λ (nm)	*n_z_*	*n_y_*	*n_x_*	χ_112_	χ_211_	χ_222_	χ_123_	χ_213_	χ_312_	χ_233_	χ_323_		
C1	∞	1.597	1.516	1.506	1.4	1.4	0.8	−0.2	−0.2	−0.2	−1.2	−1.2		
	1064	1.606	1.524	1.514	2.0	1.9	1.0	−0.3	−0.2	−0.3	−1.4	−1.4		
	532	1.638	1.553	1.543										

## Data Availability

Crystallographic data can be obtained free of charge as supporting information and also via the CCDC at https://www.ccdc.cam.ac.uk/data_request/cif, by emailing data_request@ccdc.cam.ac.uk or by contacting The Cambridge Crystallographic Data Centre: CCDC entries 2380328, 2380329, 2380335 and 2380336 contain the supporting crystallographic data for this paper.
