# Additive-driven microwave crystallization of tyramine polymorphs and salts: a quantum crystallography perspective. Corrigendum

**DOI:** 10.1107/S2052252526003283

**Published:** 2026-05-18

**Authors:** Szymon Grabowski, Klaudia Nowakowska, Helena Butkiewicz, Anna Hoser, Aleksandra Wesełucha-Birczyńska, Tomasz Seidler, Paulina Moskal, Marlena Gryl

**Affiliations:** a Faculty of Chemistry Jagiellonian University Gronostajowa 2 Krakow30-387 Poland; b Doctoral School of Exact and Natural Sciences Jagiellonian University Prof. St. Łojasiewicza 11 Krakow30-348 Poland; c Biological and Chemical Research Centre Faculty of Chemistry University of Warsaw Żwirki i Wigury 101 Warsaw02-089 Poland

**Keywords:** tyramine, polymorphism, microwave cocrystallization, electron density studies, optical properties, corrigendum

## Abstract

Support by National Science Centre Poland under grant No. UMO-2023/51/B/ST5/02843 for the work described in the article by Grabowski *et al.*[(2025), *IUCrJ*, **12**, 403–416] is acknowledged.

## Abstract 2

The article by Grabowski *et al.*[(2025), *IUCrJ*, **12**, 403–416] is corrected.

Grant No. UMO-2023/51/B/ST5/02843 from National Science Centre Poland was not acknowledged in the article by Grabowski *et al.* (2025) ▸. The full funding statement is given below.

## References

[bb1] Grabowski, S., Nowakowska, K., Butkiewicz, H., Hoser, A., Wesełucha-Birczyńska, A., Seidler, T., Moskal, P. & Gryl, M. (2025). *IUCrJ*, **12**, 403–416. 10.1107/S2052252525002210 PMC12044854 40293196

