# Supplementary material for: Additive-driven microwave crystallization of tyramine polymorphs and salts: a quantum crystallography perspective
Source: IUCrJ. 2025 Apr 28;12(Pt 3):403–16. doi: 10.1107/S2052252525002210 (PMC12044854; doi:10.1107/S2052252525002210)
Supplement: Supplementary file 6 [file m-12-00403-sup6.pdf]

IUCrJ

Volume 12 (2025)

Supporting information for article:

Additive-Driven Microwave Crystallization of tyramine polymorphs and salts: a quantum crystallography perspective

Szymon Grabowski, Klaudia Nowakowska, Helena Butkiewicz, Anna Hoser, Aleksandra Wesełucha-Birczyńska, Tomasz Seidler, Paulina Moskal and Marlena Gryl

Normal Mode Refinement Details

This level of theory was previously used by us for normal mode refinement and has proven to be sufficient (Sovago *et al.,* 2020). We conducted frequency calculations at the Γ point of the Brillouin zone. Prior to these calculations, the geometry was optimized, with convergence criteria set to the defaults for frequency calculations using the PREOPTGEOM keyword. Since we optimized only the atomic coordinates, the frequency calculations were carried out using cell parameters obtained from X-ray diffraction measurements. The BUNITSDECO command was used to analyze the vibrational modes, providing information on the decomposition of the building units in terms of internal and external motions, in both cases first six frequencies have high external contribution and later on we decided to refine six frequencies in normal mode refinement. Input files for the CRYSTAL17 frequency calculations can be readily generated using the cif2crystal routine (http://shade.ki.ku.dk/docs/cif2crystal/cif2crystal.html). Normal mode refinement can be conducted via server nomore.chem.uw.edu.pl (Hoser *et al*, 2021).

Additional information about crystal structures of T1 and T2

The orange chains in T1 are created from N2B-H22···O1B^{iii} hydrogen bonds and connected by stronger O1B-H1B···N2B^{i} together with weak C2B-H2B···O1B interactions and C-H···π (C2B-H2B···CG). The violet chains are similar because they are also composed of comparable in power hydrogen bonds N2A-H12···O1A^{iii}, and connected by stronger O1A-H1A···N2A^{ii} with C-H···O and C-H···π interactions (C2A-H2A···O1A and C2A-H2A···CG, respectively). The difference between orange and violet molecules is mainly in the position of the hydroxyl group with respect to the alkylamine chain (Fig. S3). In T2 the green and the blue chains are again built of moderate hydrogen bonds O1A-H1A···N2A^{i} and O1B-H1B···N2B^{i}, respectively. The pairs of chains are stabilized by weak N2A-H2A2···O1A^{ii} and N2B-H2B1···O1B^{ii} hydrogen bonds, which are bifurcated and create $R_2^2(4)$ motif, shown in Fig. S4. The blue and green molecules (Fig. S3) appear as mirror images of each other. The hydrogen bonds' strength was analysed here based on geometric parameters which can be found in Tables S2 and S3.

Table S1 Experimental data for T1, T2, C1 and C2.

**Structure**	T1	T2	C1	C2
**Crystal data**				
Chemical formula	$C_8H_{11}NO$	$C_8H_{11}NO$	$C_{32}H_{47}N_5O_7$	$C_{16.67}H_{24}N_{3.33}O_4$
M_r	137.18	137.18	613.74	335.06
space group	*P*c	$P\bar{1}$	$P2_1$	$R\bar{3}c$
Temperature (K)	100(2)	100(2)	298(2)	100(2)
a, *b*, *c* (Å)	9.1243(5), 9.6491(4), 8.7744(4)	5.4977(2), 8.9826(4), 14.6789(7)	9.1192(1), 19.0384(2), 10.0172(1)	17.2368(1), 17.2368(1), 62.5877(3),
α, β, γ (°)	90, 104.232(5), 90	85.742(4), 89.930(4), 81.520(4)	90, 95.648(1), 90	90, 90, 120
V ($Å^3$)	748.80(6)	714.96(5)	1730.69(3)	16103.9(2)
Z	4	4	2	36
Radiation type	MoK_α	MoK_α	CuKα	CuKα
μ (mm^{-1})	0.081	0.084	0.682	0.739
Crystal size (mm)	0.46 x 0.26 x 0.10	0.49 x 0.47 x 0.05	0.20 x 0.12 x 0.07	0.28 x 0.25 x 0.18
**Data collection**				
Diffractometer	XtaLAB Synergy, Dualflex, HyPix	XtaLAB Synergy, Dualflex, HyPix	XtaLAB Synergy, Dualflex, HyPix	XtaLAB Synergy, Dualflex, HyPix
Absorption correction	Multi-scan *CrysAlis PRO* 1.171.42.100a (Rigaku Oxford Diffraction, 2023) Empirical absorption correction using spherical harmonics, implemented in SCALE3 ABSPACK scaling algorithm.	Multi-scan *CrysAlis PRO* 1.171.42.100a (Rigaku Oxford Diffraction, 2023) Empirical absorption correction using spherical harmonics, implemented in SCALE3 ABSPACK scaling algorithm.	Multi-scan CrysAlis PRO 1.171.42.100a (Rigaku Oxford Diffraction, 2023) Empirical absorption correction using spherical harmonics, implemented in SCALE3 ABSPACK scaling algorithm.	Multi-scan CrysAlis PRO 1.171.42.100a (Rigaku Oxford Diffraction, 2023) Empirical absorption correction using spherical harmonics, implemented in SCALE3 ABSPACK scaling algorithm.
No. of measured, independent and observed [$I > 2\sigma(I)$] reflections	19416/6659/4613	19022/6748/5114	36090/7442/7120	188207/3914/3872
R_{int}	0.058	0.041	0.0376	0.0480
$(\sin \theta/\lambda)_{max}$ ($Å^{-1}$)	0.851	0.852	0.640	0.640
**Refinement**				
$R[F^2 > 2\sigma(F^2)]$, $wR(F^2)$, S	0.062, 0.140, 1.03	0.060, 0.174, 1.00	0.041/0.116/1.06	0.064/0.148/0.961
Data/restraints/parameters	6659/2/199	6748/0/199	7442 / 4 / 438	3914 / 2 / 232
H-atom treatment	H atoms treated by a mixture of independent and constrained refinement	H atoms treated by a mixture of independent and constrained refinement	H atoms treated by a mixture of independent and constrained refinement	H atoms treated by a mixture of independent and constrained refinement
$\Delta\rho_{max}$, $\Delta\rho_{min}$ (e $Å^{-3}$)	0.494; -0.271	0.642; -0.263	0.416; -0.188	0.309; -0.328
Absolute structure parameter	0.2(6)	-	-0.08(6)	-

Table S2 Torsion angle C6-C7-C8-N2 comparison between tyramine polymorphs

**polymorph**	**T1 (*P*c)**	**T2 ($P\bar{1}$)**
**Torsion C6-C7-C8-N2 (°)**	-175.68	178.64
	-176.39	-175.23

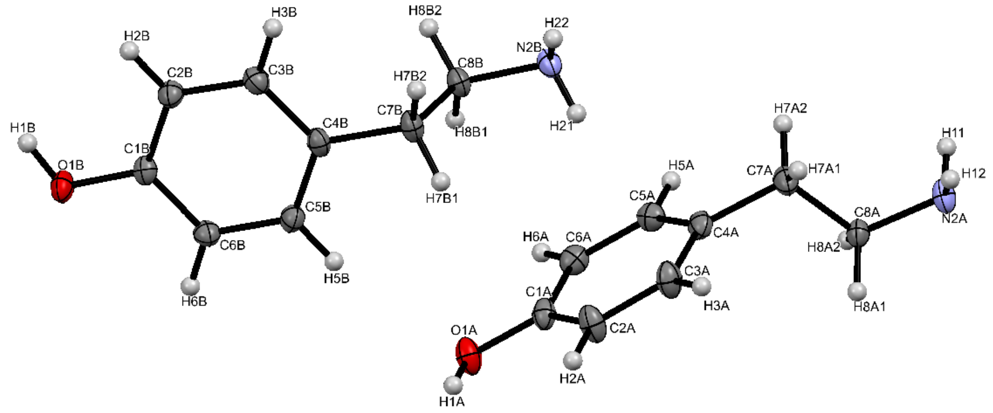

Figure S1 The contents of the asymmetric unit of T1, with the atom-numbering scheme.

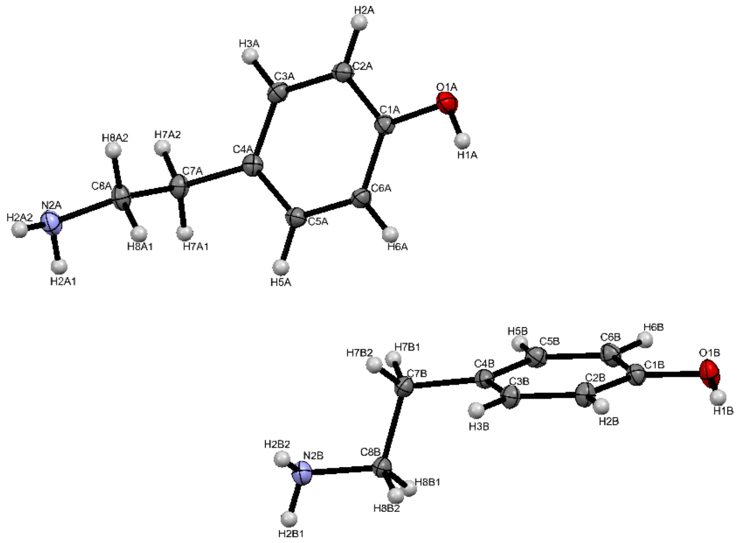

Figure S2 The contents of the asymmetric unit of T2, with the atom-numbering scheme.

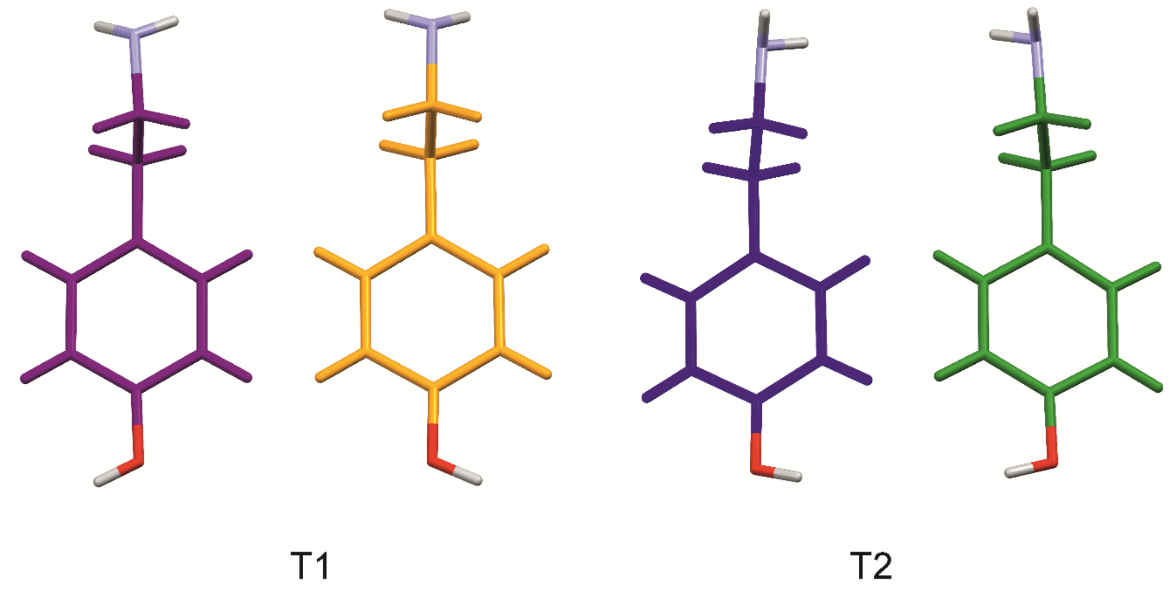

Figure S3 Tyramine molecules from T1 and T2.

Table S3 Hydrogen bonds and weak interactions in T1.

D - H···A	d(D-H) [Å]	d(H···A) [Å]	d(D···A) [Å]	<(DHA) [°]
O1B-H1B···N2B^{i}	0.89(3)	1.76(3)	2.648(2)	176(3)
O1A-H1A···N2A^{ii}	0.82(3)	1.85(3)	2.655(2)	168(3)
N2B-H22···O1B^{iii}	0.83(3)	2.21(3)	3.036(3)	177(3)
N2A-H12···O1A^{iii}	0.89(3)	2.14(3)	3.023(3)	175(3)

Symmetry codes: (i) x-1,-y,z-1/2; (ii) x-1,-y+1,z-1/2; (iii) x+1,y,z.

Table S4 Hydrogen bonds and weak interactions in T2.

D - H···A	d(D-H) [Å]	d(H···A) [Å]	d(D···A) [Å]	<(DHA) [°]
O1A-H1A···N2A^{i}	0.91(2)	1.81(2)	2.700(1)	169(2)
O1B-H1B···N2B^{i}	0.88(2)	1.84(2)	2.708(1)	170(2)
N2B-H2B1···O1B^{ii}	0.87(2)	2.65(2)	3.287(1)	131(1)
N2A-H2A2···O1A^{ii}	0.89(2)	2.64(2)	3.343(1)	137(2)
C2A-H2A···O1A^{iii}	0.95	2.63	3.401(1)	138

Symmetry codes: (i) x,y+1,z; (ii) x+1,y-1,z; (iii) -x,-y+1,-z+1.

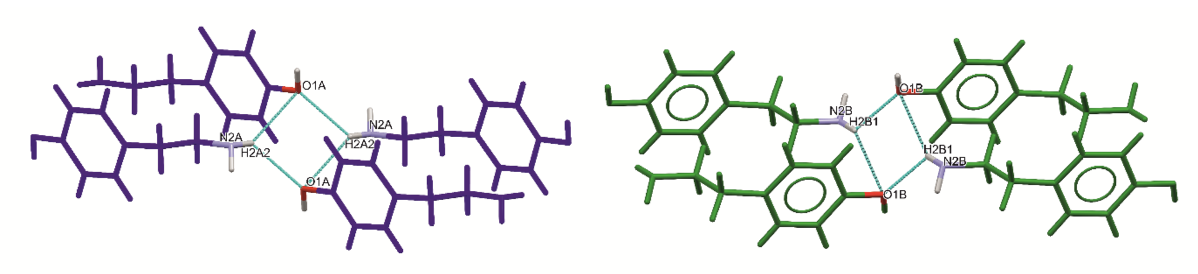

Figure S4 The $R_2^2(4)$ motif in T2 created by bifurcated hydrogen bonds.

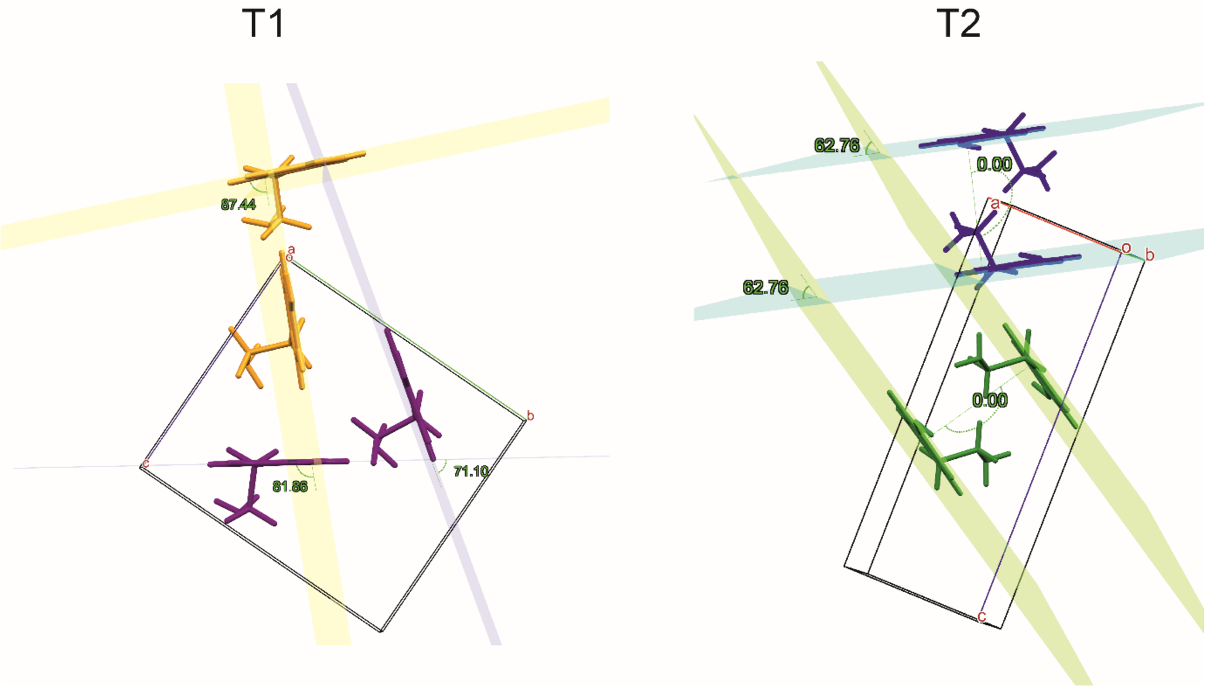

Figure S5 The angles between planes constructed on the phenyl rings in T1 and T2.

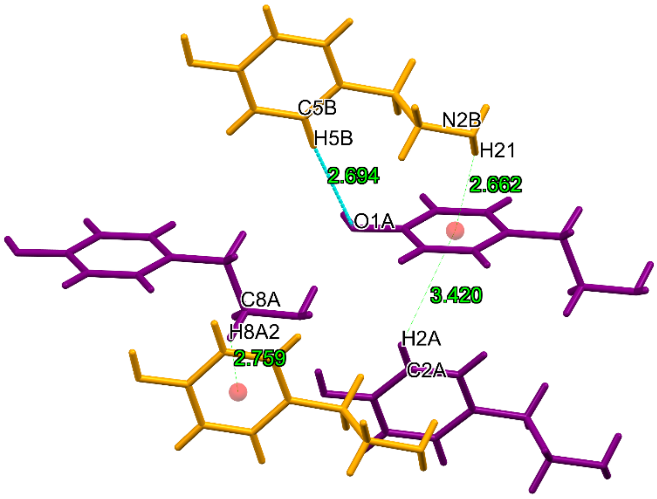

Figure S6 Weak interactions and distances in Å between hydrogen and acceptor or hydrogen and centroid in T1.

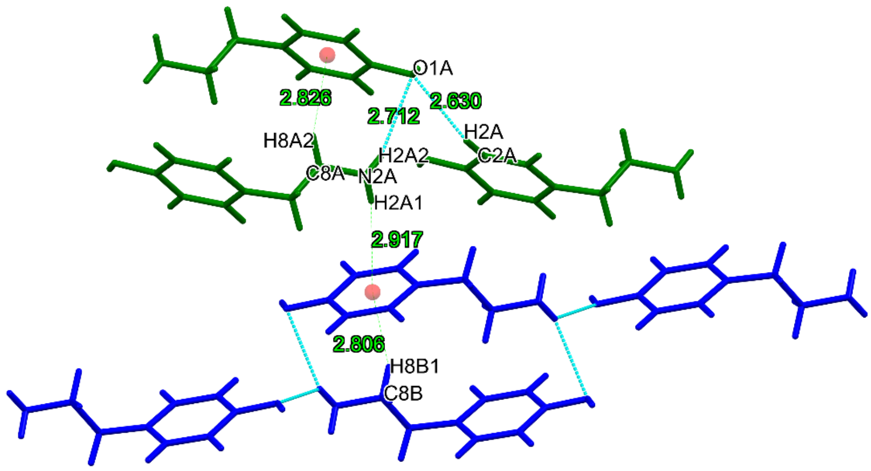

Figure S7 Weak interactions and distances in Å between hydrogen and acceptor or hydrogen and centroid in T2.

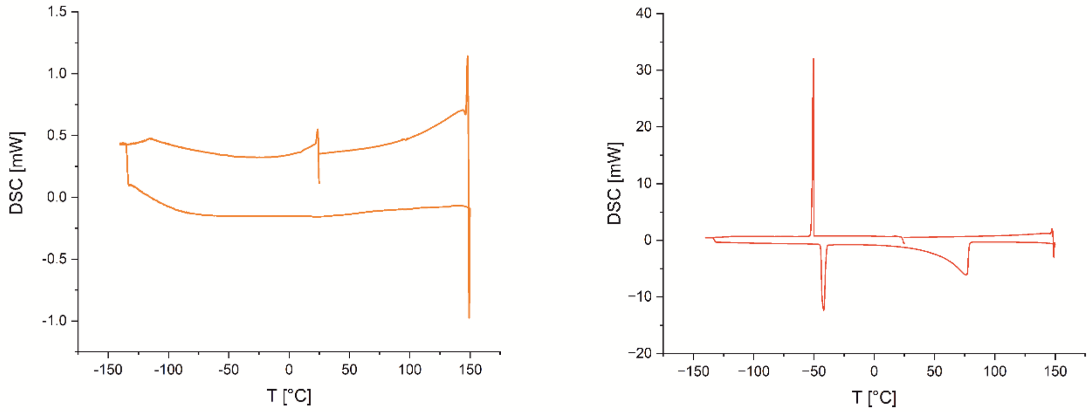

Figure S8 Differential Scanning Calorimetry curves for tyramine polymorphs T2 (orange) and T1 (red).

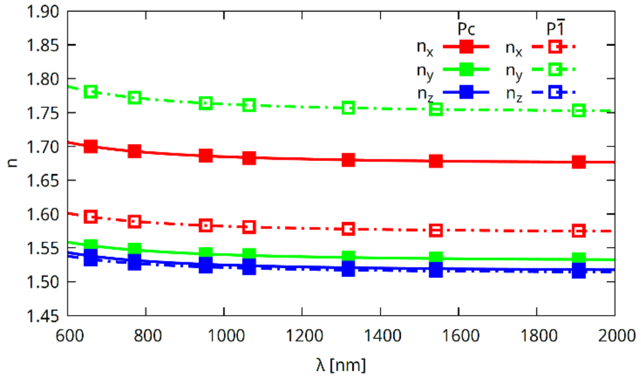

Figure S9 Dispersion of the refractive indices for T1 (Pc) and T2 (P-1).

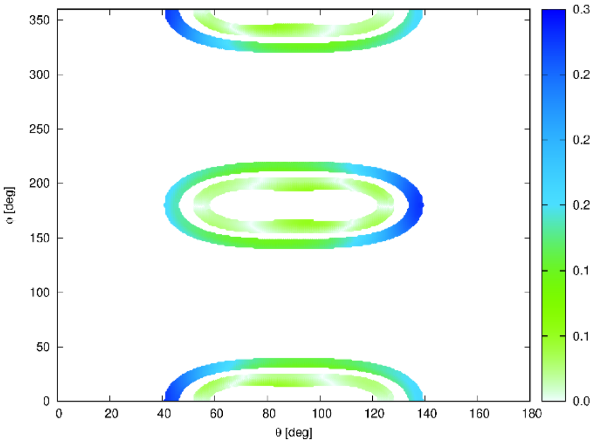

Figure S10 Theoretically predicted deff mapped on phase-matching configurations at 1318 nm (based on the MP2/Q-LFT approach results); the inner figures correspond to type I interaction while the outer figures correspond to type II interaction.

Table S5 Prominent Raman bands observed at 100 K and 300 K and their assignment.

Raman band position [cm^{-1}]		Assignment (Siddiqui *et al.*, 2009; Tu, 1982)
100 K	300 K	[P.E.D]
367	368	τ(CCCO)[56] + δ(CCC)adj N[12]
387	387	τ(HOCC)[98]
467	467	τ(CCCC)R[87
643	643	δ(CCC)R[84]
823	823	τ(HCCO)R[44] + ν(CC)adj R[16] + ν(NC)[12]
849	849 (max)	ν(CC)adj R[60] + τ(HCCO)R[13]
1088	1081	δ(HCC)adj R[50] + ν(CC)R[15] + δ(HNC)[11]
vw	1172	ν(CC)adj R[52] + δ(CCC)R[11]
1205	1205	δ(HCC)adj N[57] + δ(HNC)[13]
1257	1257	ν(OC)[67
1294	1294	δ(HCC)adj N[79] + ν(CC)R[11]
1364	vw	ν(CC)R[54] + δ(HOC)[22]
1438	1437	δ(HCH)adj R[73] + τ(HCCC)adj R[11]
1591	1591	ν(CC)R[67]
1614 (max)	1614	ν(CC)R[62] + δ(HCC)R[17]
2850	2849	νs(CH2)adj R[99]
	2869	νas(CH2)adj R[98]
2895	2892	νas(CH2)adj R[98]
2940	2939	νs(CH2)adj N[99]
3040	3040	ν(CH)R[91]
3071	3069	ν(CH)R[97]

ν: stretching; νs: symmetric stretching; νas: asymmetric stretching; τ-torsional vibrations, δ-bending vibrations; R: Ring; adj: adjacent

Table S6 Analysis of selected Raman bands intensity ratios.

Raman band ratios			Characteristics (Siddiqui *et al.*, 2009; Tu, 1982)
	100 K	300 K	
I_{367}/I_{387}	1.46±0.05	0.48±0.05	skeletal vibrations adjacent to N vs ring
I_{849}/I_{823}	2.22±0.05	2.47±0.05	ν(CC) ring τ(HOCC)
I_{1614}/I_{1591}	3.36±0.05	6.60±0.05	ν(CC) ring vibrations
I_{2869}/I_{2850}	0.30±0.05	0.51±0.05	indicators of lateral packing
I_{2900}/I_{2850}	0.41±0.05	0.78±0.05	
I_{2940}/I_{2850}	0.26±0.05	0.49±0.05	CH_2 residue adjacent to the N
I_{3040}/I_{3071}	1.21±0.05	0.72±0.05	ν(CH) aromatic

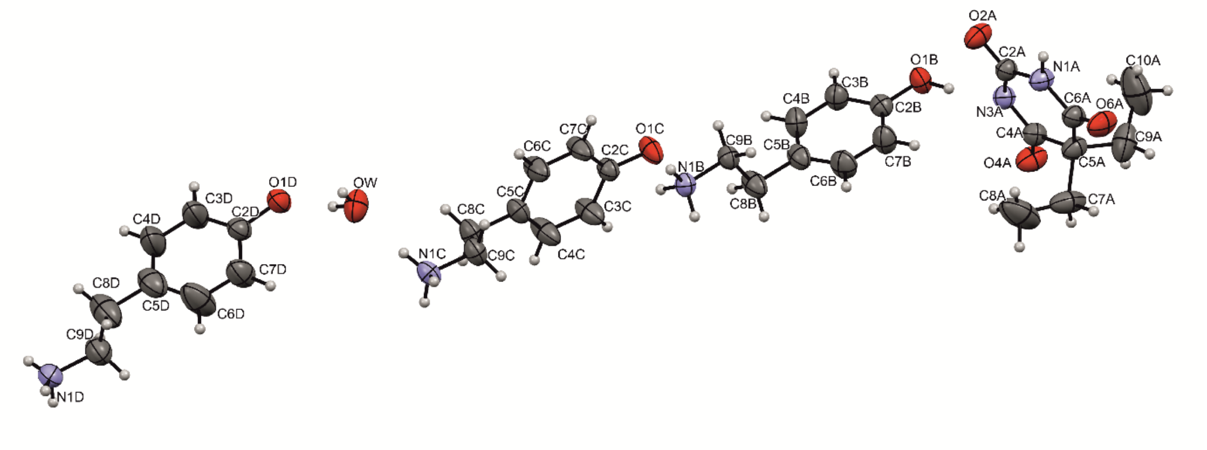

Figure S11 The contents of the asymmetric unit of C1, with the atom-numbering scheme. Displacement ellipsoids are drawn at the 50% probability level. The drawing was prepared with Mercury.

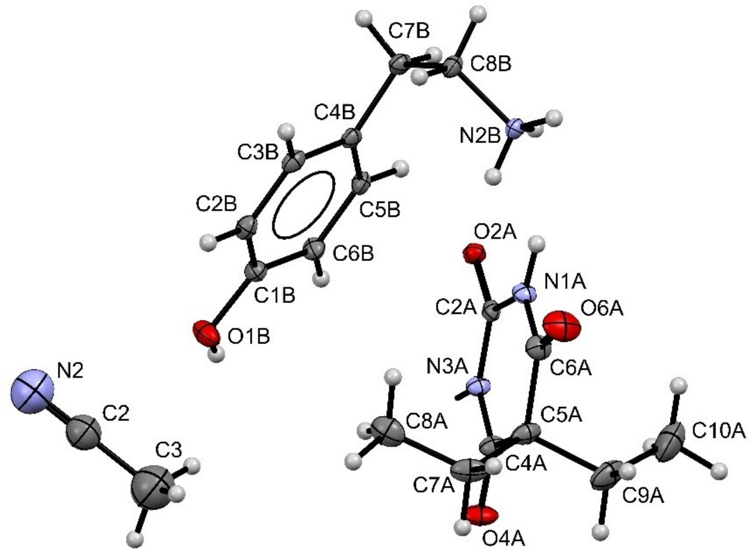

Figure S12 The contents of the asymmetric unit of C2, with the atom-numbering scheme. Displacement ellipsoids are drawn at the 50% probability level. For the sake of clarity of the drawing we disregarded the disordered solvent and included only one acetonitrile molecule.

Table S7 Hydrogen bond geometry in C1.

D - H···A	d(D-H) [Å]	d(H···A) [Å]	d(D···A) [Å]	<(DHA) [º]
N1A – H1A···O1D^{viii}	0.91((3)	1.874	2.789	175.41
N3A – H50 ···O1B	1.015	1.723	2.679	155.31
N1B – H1NB···O1B^{iv}	0.93(3)	2.351	3.046	131.39
N1B – H1NB ···O2A^{iv}	0.93(3)	2.094	2.806	132.55
N1B – H2NB···O6A^{vii}	0.98(3)	1.897	2.864	167.56
N1B – H3NB···O1C	1.04(3)	1.539	2.576	175.86
O1B – H1OB···O2A	0.94(4)	2.704	3.309	122.76
N1C – H1NC···O1C^{iv}	0.91(3)	1.775	2.678	170.72
N1C – H2NC···O4A^{vi}	0.92(3)	1.986	2.859	157.98
N1C – H3NC···OW	0.91(4)	1.867	2.769	173.98
N1D – H1ND···O2A^{i}	0.75(4)	2.660	3.124	122.41
N1D – H1ND ···O1C^{ii}	0.75(4)	2.397	3.057	148.28
N1D – H2ND···O4A^{iii}	0.87(4)	1.989	2.840	166.46
N1D – H3ND···O1D^{iv}	0.96(4)	1.658	2.615	174.52
OW – H1O···O1D	0.85(3)	1.807	2.646	171.09
OW – H2O···O6A^{v}	0.84(3)	2.429	3.196	151.33

Symmetry codes: (i) 1-x,-1/2+y,-z; (ii) -x,1/2+y,-z; (iii) 1-x,-1/2+y,1-z; (iv) -1+x,y,z; (v) 2-x,1/2+y,-z; (vi) -2+x,y,-1+z; (vii) 3-x,-1/2+y,1-z; (viii) 2-x,-1/2+y,-z

Table S8 Hydrogen bond geometry in C2.

D - H···A	d(D-H) [Å]	d(H···A) [Å]	d(D···A) [Å]	<(DHA) [º]
N1A – H1A···O2A^{i}	0.879	1.995	2.868	172.43
O1B – H1B···O4A^{iii}	0.839	1.863	2.698	173.00
N2B – H2BA···O6A^{ii}	0.909	2.016	2.783	141.25
N2B – H2BC···O2A^{i}	0.911	1.927	2.836	176.19

Symmetry codes: (i) 1-y,-1+x-y,z; (ii) 1/3+x-y,2/3-y,1/6-z; (iii) 1/3-x,2/3-x+y,1/6-z

Table S9 Details of theoretical intermolecular lattice energy calculations. All energies are in kJ/mol.

		T1	T2
bulk	Ebulk +D3	-4634391.63033	-4634399.64004
molecule 1	Emol1 nod +D3	-1158393.83035	-1158392.06546
	Emol1 nod	-1158301.21222	-1158299.71171
	Emol1 cpc	-1158341.34744	-1158343.24005
	BSSE1	40.13522	43.52834
molecule 2	Emol2 nod +D3	-1158392.43451	-1158391.70719
	Emol2 nod	-1158299.72322	-1158299.33392

BSSE	Emol2 cpc	-1158340.36651	-1158343.26666
	BSSE2	40.64329	43.93275
	E coh	-204.77515	-208.02368
	E coh +BSSE	-164.38590	-164.29314
